# Reproductive stage physiological and transcriptional responses to salinity stress in reciprocal populations derived from tolerant (Horkuch) and susceptible (IR29) rice

**DOI:** 10.1038/srep46138

**Published:** 2017-04-11

**Authors:** Samsad Razzaque, Taslima Haque, Sabrina M. Elias, Md. Sazzadur Rahman, Sudip Biswas, Scott Schwartz, Abdelbagi M. Ismail, Harkamal Walia, Thomas E. Juenger, Zeba I. Seraj

**Affiliations:** 1 Plant Biotechnology Lab, Department of Biochemistry and Molecular Biology, University of Dhaka, Dhaka, 1000, Bangladesh; 2 Department of Integrative Biology and Institute for Cellular and Molecular Biology, University of Texas, Austin, Texas 78712, USA; 3 Department of Agronomy and Horticulture, University of Nebraska, Lincoln, Nebraska 68583, USA; 4 Plant Physiology Division, Bangladesh Rice Research Institute, Gazipur, Bangladesh; 5 International Rice Research Institute, Los Banos, Laguna, Philippines

## Abstract

Global increase in salinity levels has made it imperative to identify novel sources of genetic variation for tolerance traits, especially in rice. The rice landrace Horkuch, endemic to the saline coastal area of Bangladesh, was used in this study as the source of tolerance in reciprocal crosses with the sensitive but high-yielding IR29 variety for discovering transcriptional variation associated with salt tolerance in the resulting populations. The cytoplasmic effect of the Horkuch background in leaves under stress showed functional enrichment for signal transduction, DNA-dependent regulation and transport activities. In roots the enrichment was for cell wall organization and macromolecule biosynthesis. In contrast, the cytoplasmic effect of IR29 showed upregulation of apoptosis and downregulation of phosphorylation across tissues relative to Horkuch. Differential gene expression in leaves of the sensitive population showed downregulation of GO processes like photosynthesis, ATP biosynthesis and ion transport. Roots of the tolerant plants conversely showed upregulation of GO terms like G-protein coupled receptor pathway, membrane potential and cation transport. Furthermore, genes involved in regulating membrane potentials were constitutively expressed only in the roots of tolerant individuals. Overall our work has developed genetic resources and elucidated the likely mechanisms associated with the tolerance response of the Horkuch genotype.

The influence of abiotic stresses are responsible for an estimated decline of 50–70% in crop production worldwide. Among these abiotic stresses, salinity is considered as one of the main environmental constraints to agricultural productivity. About 20% of cultivated lands are affected (~45 million ha) by increasing salinity^1^ worldwide. The situation is especially dire in low lying and densely populated developing countries like Bangladesh. The coastal area affected by salinity in Bangladesh has increased from 0.83 to 1.06 mha during the period from 1973 to 2009^2^ and this rise is considered as a serious threat to rice (*Oryza sativa*) production because of the limited arable land in Bangladesh. The worst affected areas are in the Southwest, in Satkhira and Khulna immediately north of the Sundarbans^3^. The latter areas are however home to rice landraces, which can likely serve as sources of salt tolerance for rice breeding programs.

Rice is the most important cereal crop but is highly salt-sensitive among major cereals. This sensitivity is variable at different stages of its growth period. Rice plants are most sensitive at the young seedling and reproductive stages^4^ but relatively tolerant at seed germination^5^. Moreover, seedling and reproductive stage tolerances are poorly correlated, suggesting separate sets of genes may be involved at different developmental stages in overcoming salt stress^6^,^7^. There are a number of seedling stage gene expression studies in rice but few^8^ at the reproductive stage. At the reproductive stage, spikelet fertility and numbers per panicle are reported to be the most affected yield components under salt stress^9^. A better understanding of the mechanism of salt tolerance in rice at the reproductive stage is a necessary step in efforts to improve grain set under salt stress.

Some genetic mechanisms of salinity tolerance in cereal crops have been identified and include genes like NHX^10^, SOS1^11^, HKT^12^, NAC^13^, bZIP^14^, Hardy^15^, PDH45^16^, glyoxalases^17^, chaperones^18^ and various antioxidants that respond to salinity stress at the transcriptional level. The identification of these genes has been primarily through their sequence similarity with proteins of known functions in salt tolerance in model species. Some of these genes have been reported to play important roles in protecting plants from salinity stress though perception, signal transduction and transcriptional regulatory networks in cellular responses^19^. Some understanding of the pathways involved in salt stress response in plants has been made, such as those involved in ion transport and homeostasis, osmoregulation, and oxidative stress protection^20^. These responses have been broadly categorized into three different mechanisms including, for example, growth reduction, ion exclusion, and tissue tolerance, and usually one or more of these pathways are involved^21^. Nevertheless, these studies have not yet provided a global understanding of salinity-induced physiological responses, particularly in rice genotypes that span broad natural variability in salinity tolerance. This in turn has led to the suggestion for utilizing uniquely stress “adapted” genotypes to study the mechanism of salt tolerance^22^.

In salt-affected areas, farmers are known to have adopted the use of rice landraces for generations even though these generally have poor agronomic traits including tall plant stature, long growth duration, low yield, and poor grain quality^23^. These traditional varieties however show significant tolerance to salt stress by a complex set of physiological mechanisms which include sodium exclusion, shown in Pokkali and Nona Bokra^24^ compartmentation into vacuoles in Pokkali^25^ or older leaves as in Horkuch^26^ as well as stomatal responsiveness again found in Pokkali^27^. Upregulation of metallothionein-like protein were also reported both for Pokkali^9^ and Horkuch^26^. Metallothionein which is a small molecular weight cysteine-rich protein has been reported to confer salt tolerance though efficient scavenging of ROS in tobacco. There is also upregulation of the antioxidant machinery and other necessary genes including transcription factors (TF) associated with salt tolerance in a number of rice genotypes^28^. However, most tolerant rice landraces seem to possess only a subset of these traits. Therefore, it has become important to identify specific characteristics in different genotypes, leading to the possibility of recombining these tolerance traits into a single high-yielding variety.

In this study we used a population derived from the salt-tolerant landrace popular among farmers, Horkuch, which is endemic to the southern coast of Bangladesh. Horkuch was previously shown to have less reduction in shoot biomass, low Na^+^: K^+^ ratio in flag leaf, less reduction in yield and good partitioning of Na^+^ in the older leaves as well as high levels of Ca^2+^ and Mg^2+^ in the flag leaves when subjected to salinity stress at reproductive stage^26^. These data suggested a polygenic trait likely regulated by multiple physiological mechanisms. The genetic factors underlying these mechanisms remain to be discovered within the known salt-tolerant landraces. An ideal strategy for accomplishing these goals would be to recombine these variations in genetic mapping populations, followed by phenotyping and analyzing RNA transcripts to disentangle the expression variation associated with tolerance versus sensitivity. In the current work, reciprocally crossed populations were used for RNAseq analyses because transcriptional variation has been observed before in reciprocal hybrids as well as their inbred parents^29^. Moreover, earlier work indicated the beneficial effect of the chloroplast photosystem components in Horkuch^26^. Our aim was to identify genes involved in response to salinity stress by RNAseq analysis using a novel 3ʹtag-seq method in the leaf and root tissues resulting in the tolerance (high 1000 GW) or sensitive (low 1000 GW) response of F_3_ plants at the reproductive stage^30^,^31^. We categorized tolerant and sensitive plants from the reciprocal populations based on thousand grain weight (THGW) and then studied gene expression in those in a separate experiment in both leaf and root tissues under non-saline and salinity stress condition. We observed significant differences in stress related expression between tissue types and cross direction. We detected a number of significant genes and their associated functional enrichments in tolerant plants that varied from sensitive progenies.

## Results

### Physiological effects of stress on yield-related traits in sensitive and tolerant progenies

**Figure 1:**
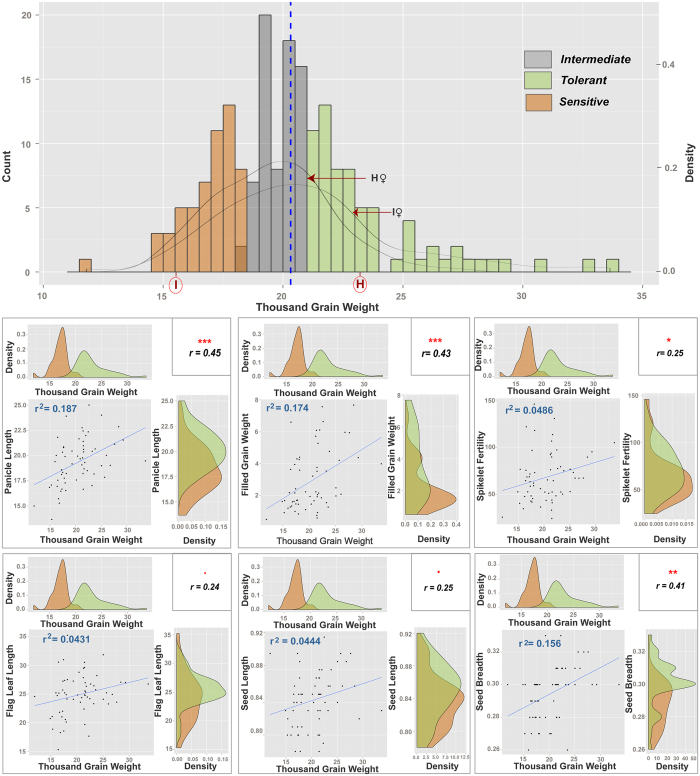
Phenotypes of the reciprocal population and selected sensitive and tolerant progenies under stress. The top panel shows distribution of the whole population from both reciprocal crosses based on thousand grain weight phenotype. Selection of tolerant, sensitive and intermediate plants are shown in color. The bottom panel shows correlation of thousand grain weight with six other yield related traits, i.e. Panicle Length (P.L.), Filled grain weight (F.G.W.), Spikelet fertility (S.F.), Flag leaf length (F.L.L.), Seed length (Se L.) and seed breadth (Se B.). Corresponding distribution curves of selected tolerant and sensitive progenies are also shown for each trait. The correlation values (r), regression values (r^2^) and their significance level are displayed in the accompanying boxes on top right of each segment.

**Figure 2:**
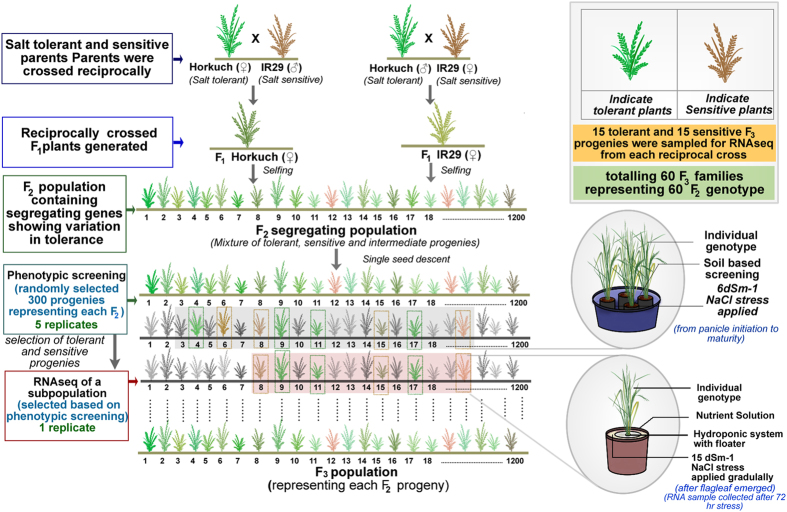
Schematic representation on the overall experimental design for phenotype selection and sampling in the RNAseq study. The picture shows a step by step procedure from crossing events to collecting samples for RNAseq study.

We measured the 1000 GW (THGW) of the reciprocal F_3_ populations (Fig. 1 (Detailed experimental procedures have been described in the Method section)). Both the reciprocal populations show a normal distribution in THGW. The basis of the selection of tolerant and sensitive progenies has been explained in details in ‘categorization of sensitive and tolerant plants at reproductive stage’ in the methods section. The tolerant and sensitive progenies based on THGW are shown in the Fig. 2 in green and light brown, respectively. Based on this experiment, Horkuch falls in the tolerant and IR29 is in the sensitive category. Fifteen replicates from the tolerant and sensitive genotypes were selected for RNAseq analysis (Supplementary Dataset 1). The selected tolerant and sensitive progenies are indicated in the 2^nd^ panel in Fig. 1, where these are clearly separated. Therefore, use of THGW to determine the sensitive genotypes from the tolerant genotypes was found to be adequate and justified. Moreover, other yield-related traits of the separated sensitive and tolerant progenies, such as panicle length (PL), filled grain weight (FGW), spikelet fertility (SF), flag leaf length (FLL), seed length (SeL) and seed breadth (SeB) correlated with THGW. PL, FGW and SeB showed a highly significant (P < 0.001) correlation with THGW (lower panels in Fig. 1).

### Transcript abundance is highly correlated within tissue types

**Figure 3:**
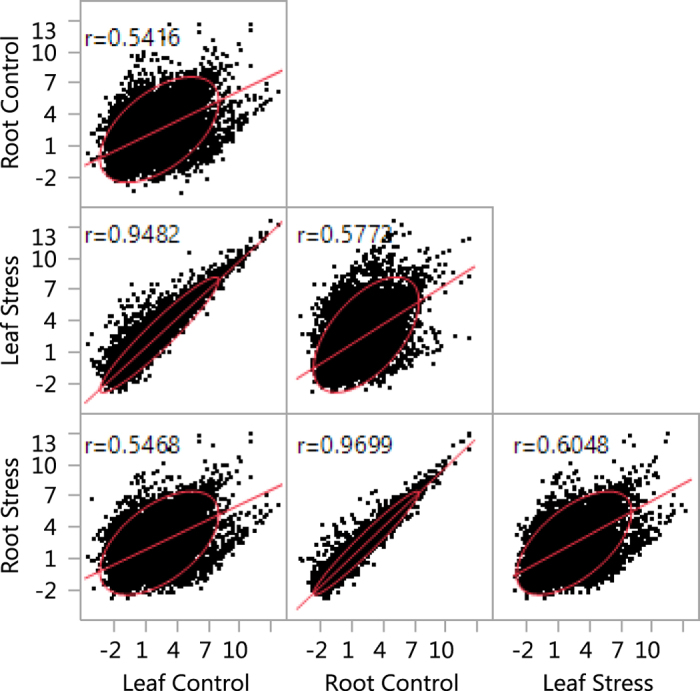
Correlation graph generated from the overall transcript counts of leaf and root tissue under control and stress condition. The graph shows here a strong correlation within same tissue type (94–96%) but comparatively poorer correlation (50–60%) between different tissue types.

We evaluated the overall pattern of transcript variation across leaf and root tissue samples using a straightforward transcript-by-transcript correlation analysis. There are large differences in transcript expression between leaf and root tissues in both control and stress conditions. The genome-wide expression correlation coefficient in control samples between leaf and root was only 0.54 (p < 0.0001) and 0.6 (p < 0.0001) when we compared leaf and root tissue under stress. However, the correlations coefficients between transcripts expressed within either the leaf or root transcriptome under the contrasting treatments was high, on average 0.94 for leaf control vs leaf stress and 0.96 for root control vs root stress (Fig. 3). Given these strikingly different patterns of expression, we have analyzed leaf and root tissues separately in all subsequent analyses.

### Number of differentially expressed genes from fixed model and pairwise interactions

**Figure 4:**
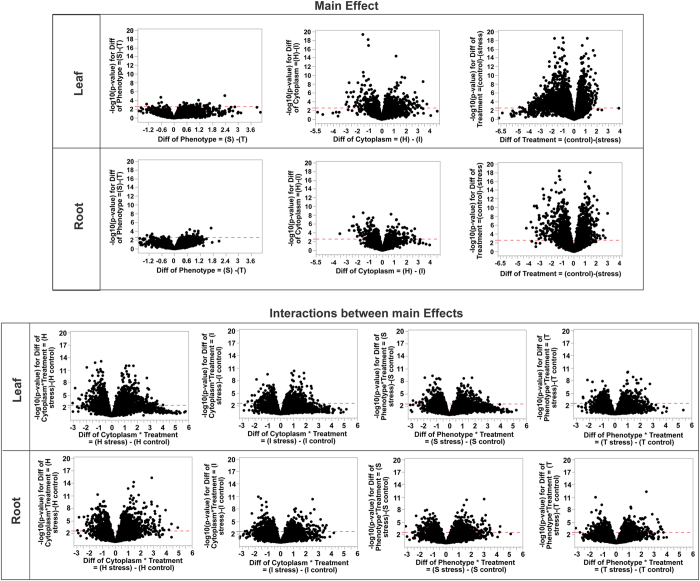
Volcano plots of significant genes in leaf and root tissue from main factors including cytoplasm, treatment, phenotype and their interactions after RNAseq analysis. The x-axis represents the natural logarithm of fold change (Fc) and the y-axis represents log10 of the P-value of each gene. The figure is separated by their main effects and the interaction effects. Main effects and interaction effects are scaled separately.

Differentially expressed genes (DEGs) were identified using with fixed effects (factors of the experimental design) and their pairwise interactions. Significant DEGs were observed for all model factors (Table 1, Fig. 4) and in both tissue types, with a stronger DEG signal in leaf tissue. We observed an especially robust signal for the main effect of salinity treatment (2201 genes in leaf, 1481 genes in roots) and interactions between cross direction and treatment (1730 genes in leaf, 1035 genes in roots from Horkuch♀ and 915 genes in leaf, 526 genes in root from IR29♀ population). In contrast, a relatively small number of DEGs were detected based on salinity tolerance phenotype. Here, only 30 and 20 genes were detected for leaf and root tissue, respectively. The interactions between phenotype and treatment detected higher number of genes in sensitive plants (656 genes in leaf, 463 genes in roots) compared to tolerant plants (411 genes in leaf and 366 genes in roots) (Table 1, Fig. 4).

In general, we observed higher differential expression in leaves vs. roots. This included both upregulation and downregulation of gene expression across our experimental factors. For example, we detected 1399 and 816 upregulated genes in leaf and roots respectively, in response to the salinity treatment. Similarly, we detected 802 and 665 genes downregulated in leaves and root, respectively. In this context, it was interesting to note that tolerant plants showed an opposite trend. A higher number of both downregulated and upregulated genes (173 and 11 respectively) in roots and compared to leaf tissues (139 and 9 respectively) were observed. In all other cases there were higher number of genes both in the upregulated and downregulated genes lists in leaf tissues. We have also observed an interaction between cross direction and treatment, in that more genes were detected in the Horkuch♀ population compared to IR29♀ population (Fig. 4). In the IR29♀ population total detected DEGs were 48% lower in leaf and 50% lower in root compared to those detected in the Horkuch♀ population. (Table 1).

Overall, our highly replicated experimental design was powerful and allowed the detection of relatively subtle differential expression. For example, we were able to detect genes as significantly differentially expressed at FDR of 0.05 with <2 fold-change. In general, fold-changes ranged from −4 to 7 (log scale) in both leaf and root tissue. We saw big differences in expression related to treatment, but smaller expression differences associated with tolerance phenotype (Table 1, Fig. 4). The range of expression variations of differentially expressed genes in sensitive plants was much larger than the tolerant plants in leaf tissues and only slightly varied in root tissues (Figs 3 and 4).

### Functional enrichments analyses show different roles between tissue types

**Figure 5:**
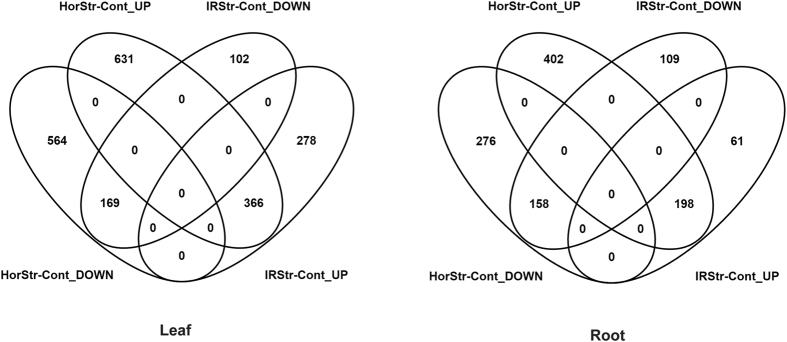
Venn diagram shows the number of genes and overlaps from the pairwise interactions of cross direction × treatment factors in leaf and root tissues. Horkuch♀ population has higher numbers of DE genes compared to IR29♀ population in both tissues.

We further investigated the DEGs identified from the major effects of the study including cytoplasm (Horkuch♀ vs IR29♀) and the treatment (control vs stress) for a wider view of the tissue-specific patterns. Specifically, differentially expressed genes discovered from these main effects were studied to see whether functional enrichments varied from leaf to root tissues. In the case of leaf and root tissue cytoplasmic effects, different classes of gene enrichments were observed. In leaf, 129 significant GO names were identified whereas in root samples 29 significant GO names were found. In leaf samples most of the enrichments were in cellular ketone metabolism, cellular amine metabolism, protein transport, defense response, response to stimuli, etc. (Supplementary Info, Fig. 5). Root GO enrichments were in defense response, programmed cell death, response to stimuli, death etc (Supplementary Info, Fig. 5). The comparative DEGs generated under treatment (stressed plants vs control plants), also indicated different classes of GOs in leaf and root tissues. The significant GO categories for leaf and root tissues under treatment effect were 201 and 123 respectively. In leaf tissues, monovalent inorganic cation transport, photosynthesis, response to water, cellular component biogenesis and signal transmission were enriched (Supplementary Info, Fig. 5). In root tissues, small molecule biosynthesis, response to chemical, negative regulation of catalytic activity, DNA conformation change, cellular carbohydrate metabolism and response to stimuli were enriched (Supplementary Info, Fig. 5). From this test it is evident that leaf and root tissues have major differences not only through transcript numbers and fold changes but also by functional enrichment tests.

### Expression analyses reveal strong signal for cytoplasmic effects

To study differential gene counts, we fitted a simple generalized linear mixed model for each transcript. This analysis modeled the normalized counts using a negative binomial distribution with a log link function. This is basically an analysis which looks row by row, at each transcript, and tests how features of our design impact number of counts. The model fitted treatment (saline and non-saline condition), cross direction (Horkuch and IR29), phenotype (tolerant and sensitive) and their interactions. The basic model we fitted looks like the following: Expression = constant + Treatment + Cytoplasm + Phenotype + Time * Treatment + Time * Cytoplasm + Treatment * Cytoplasm + Lane + Error. Here, Lane and Error are considered random effects.

The population we studied is derived from reciprocal crosses, with progenies from contrasting cross directions differing in plastid genomes (both chloroplast and mitochondria) as well as cytoplasmic environments. Both populations contributed to tolerant and sensitive progenies (Fig. 2), so studying the cross direction × treatment effect would allow us to obtain a global picture of gene expression variations and functional enrichments influenced by differences in cytoplasmic inheritance. Our experimental design included reciprocal crosses that utilized both Horkuch and IR29 as seed parents. Statistical models including Cytoplasm and Treatment*Cytoplasm resulted in identification of DEgenes depending on the main effect of cytoplasm, as well as how the effect of the salinity treatment is modified by the cytoplasmic background. We detected strong cytoplasmic influences on DEgenes in both leaf and root tissues and their associated functional enrichment studies. We obtained stronger signals of gene expression in leaf tissue compared to root when cytoplasm was considered as the main or interacting effect. We found that more genes were upregulated in the IR29 (♀) population compared to the Horkuch (♀) ones. But when we considered the interaction associated DEGs, we detected more DEgenes from the Horkuch (♀) population than the IR29 (♀) one, in both leaf and root tissues (Table 1). When we studied the functional enrichment we observed significantly altered functions in gene sets discovered from the main models and their interactions (Supplementary Info, Fig. 3). For example, the number of photosynthesis related genes from leaf tissues discovered in the Horkuch (♀) population is higher than the IR29 (♀) one similar to the response under stress. But IR29 (♀) progenies also showed higher number of differential genes enriched for localization, signaling, methylation and ion transport, etc. compared to the Horkuch (♀) ones. In root tissues, Horkuch (♀) progenies had higher amount of DEgenes associated with sulfur amino acid biosynthesis, response to chemicals, localization, response to stimuli and biological regulation in comparison to the IR29 (♀) population. However, the IR29 (♀) progenies also had some enriched functional enrichments compared to the Horkuch (♀) ones, such as, response to water and ion transport, etc. (Supplementary Info, Fig. 3). Therefore, it is clear that different sets of genes and their functional enrichments give very strong signals of cytoplasmic effects on expression profiling in our studies.

### Comparison of unique DEGs based on the sensitive and tolerant phenotype

We were interested in the DEGs present in the sensitive and tolerant progenies. Here, DEGs were found to be tissue-specific: we discovered 30 DEGs for leaf and 20 DEGs for root gene expression, with only one gene occurring in both tissue types (Table 1 and Supplementary Dataset 2). We looked at the unique genes in detail due to the low number of DEGs between the two phenotypes. GO annotation of the leaf genes indicated that these were related to DNA repair, regulation of DNA replication, cell redox homeostasis, histidine biosynthesis and cellular amino acid biosynthesis and were upregulated only in sensitive progenies. The genes involved in these biological processes were respectively, DNA repair protein Rad51, PCNA-putative DNA replicative polymerase clamp, peroxiredoxin and cystathionine gamma synthase (Accession numbers in Supplementary Dataset 2). On the other hand, in the roots of tolerant plants, the biological processes that were upregulated included response to multiple abiotic stresses, all of them involving the OsRCI2-10 hydrophobic protein LTI6A (LOC_Os07g44180), which is associated with regulation of membrane potential. The other gene upregulated in tolerant roots included the SKP1-like protein 1B involved in the Ubiquitin-dependent catabolic process. In sensitive roots, only a protein kinase domain-containing protein was upregulated involving amino acid phosphorylation (Supplementary Dataset 2 and MSU rice annotation 7).

### Comparison of unique genes from cross direction and treatment factors

**Figure 6:**
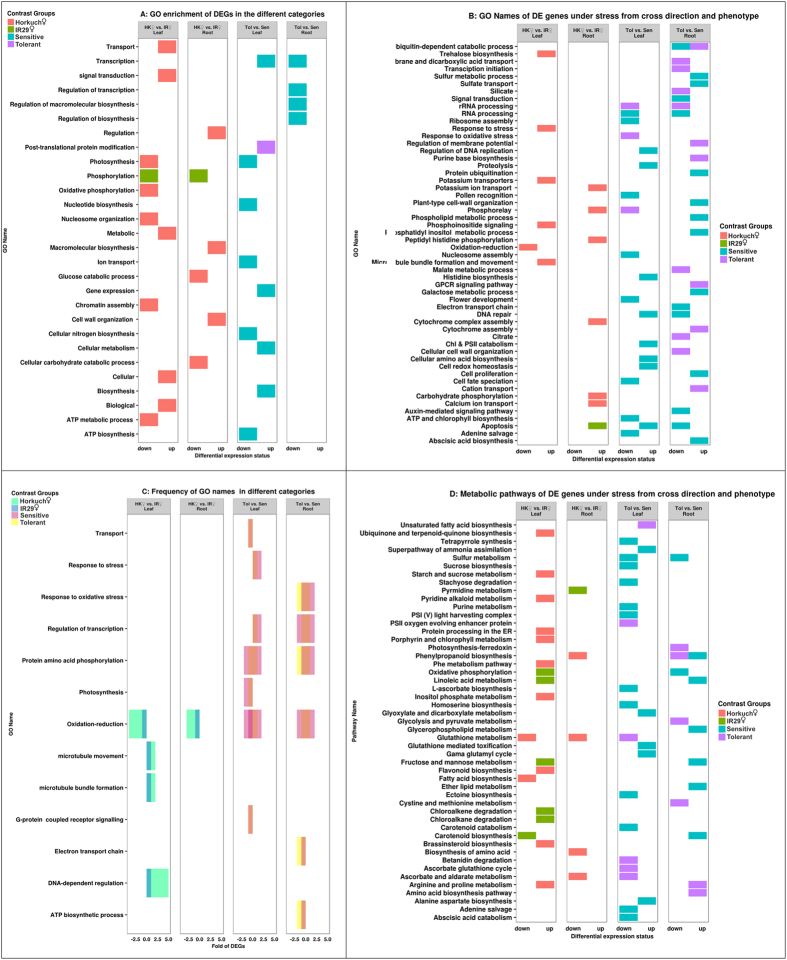
A graphical representation of GO enrichments in fixed effects and interaction factors. (**A**–**D**) DEGs from phenotype, phenotype × treatment, and cross × treatment was subjected to GO enrichment and GO annotation. GO enrichment in contrasting genotypes is in (**A**). GO names of the DEGs of contrasting genotypes were compared and their pattern of expression (up or down-regulation, root or shoot) is shown in (**B**). The number of times GO names appeared when contrasting genotypes were compared is given in (**C**). Metabolic pathways associated with these GO names is shown in (**D**). Details of the DEGs, their selection for comparison, associated common and unique GO names and their pathways is provided in Supplementary Dataset 3.

Interestingly, the number of treatment responsive DEGs discovered was almost twice that in the Horkuch♀ progenies compared to the IR29♀ ones in both leaf and root tissues (Fig. 5). We used the unique upregulated and downregulated genes from the cross direction × treatment category for GO enrichment test (Fig. 6A). GO enrichment indicated that leaves of Horkuch♀ progenies responded to stress by adjustments via signal transduction, DNA-dependent regulation and consequential increase in transport activities. The number of up and down-regulated genes associated with different GO names was mostly a few-fold higher in this population compared to reciprocal IR29♀. This included DNA-dependent regulation and a larger number of unique potassium transporters in the Horkuch♀ population compared with the reciprocal IR29♀ one (Fig. 6B and C and Supplementary Dataset 3). At the same time, Horkuch♀ progenies adjusted to stress by slowing nucleosome organization and chromatin assembly as inferred from their differential expression. Concomitantly there is a lowering of photosynthetic activity and ATP generation at 72 h, which could be a defensive action for protection against photo-oxidative damage. GO names under the corresponding down and upregulated genes are shown in Fig. 6B and the associated metabolic pathways have been depicted in Fig. 6D. The roots of the Horkuch♀ population also showed adjustment to stress perturbation by GO enrichment in cell wall organization and macromolecular biosynthesis (Fig. 6A).

In leaves of the IR29♀ population, there seems to be no coordinated adjustment in response to stress since upregulated DEGs did not show any GO enrichment. Moreover, enrichment in downregulation of the phosphorylation process indicating a lack of signaling. In roots, only the apoptosis process was enhanced while phosphorylation was downregulated, again indicating either controlled death or a lack of response to stress.

### Comparison of unique DEGs in sensitive and tolerant population under treatment

**Figure 7:**
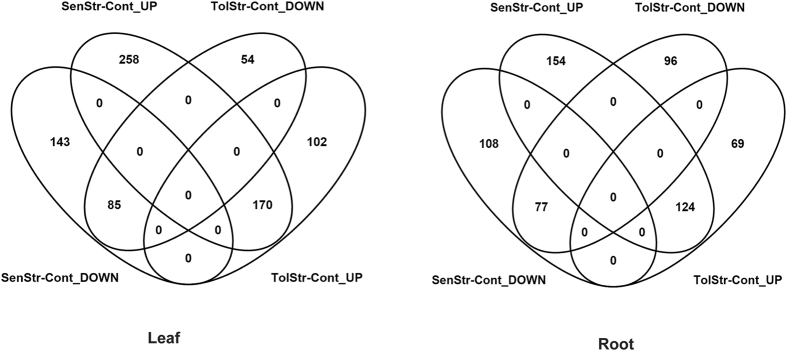
Venn diagram shows the number of genes and overlaps from the pairwise interactions of phenotype × treatment factors in leaf and root tissues. Sensitive plants have higher numbers of unique DE genes in both leaf and root tissues compared to tolerant plants.

Our primary objective was to identify gene expression associated with the sensitive and tolerant phenotype under stress. Therefore, we searched for genes showing differing responses to the treatment based on their sensitive/tolerance phenotype (Fig. 7). Our goal was to identify and differentiate (if any) groups of genes uniquely expressed under stress treatment in the sensitive versus the tolerant categories.

Salt stress had a more marked effect on the sensitive population compared to the tolerant ones. The number of up- or down-regulated DEGs in sensitive progenies was higher in both leaves and roots in comparison with the tolerant group (Supplementary Dataset 3). However, there were some exceptions where genes associated with GO names like G-protein coupled receptor signaling, transport (in leaves) and ATP biosynthesis and oxidative stress (in roots) of tolerant progenies were downregulated by greater frequency compared to the sensitive group (Fig. 6B and C). The response to stress in leaves of tolerant plants seemed subtler with GO enrichment of upregulated DEGs found only in post translational protein modification (Fig. 6A). This upregulation was in turn associated with fatty acid biosynthesis (Fig. 6D). Certain unique GO names were found to be downregulated in leaves of tolerant plants like phosphorelay, rRNA processing and response to oxidative stress. The GO names, G-protein coupled receptor signaling pathway, aquaporins and cation transport were however uniquely upregulated in roots of tolerant plants (Supplementary Dataset 3). In concurrence with our findings, showed in detail in Fig. 6A–D, Shankar *et al*.^32^ also report novel transcriptional enhancement in post-translational modification, amino acid transport and metabolism and signal transduction for the tolerant rice Pokkali. Interestingly a putative endomembrane Na^+^/H^+^ co-transporter (LOC_Os05g02240) is associated with the cation transport (MSU Release 7). Also a stress responsive protein Osr40g2 is associated with this G-protein coupled receptor protein signaling pathway (MSU Release 7). This protein was first described by Moons and coworkers^33^ in roots of the salt tolerant rice landrace Nona Bokra. On the other hand, the potassium channel protein AKT1 was observed to be associated with downregulation of ion transport in tolerant roots.

Concomitant with the higher number of DEGs in the sensitive population in comparison with the tolerant ones, there were several GO enrichments in both leaves and roots of the former (Fig. 6A). Sensitive leaves were enriched in multiple GO processes from transcription to biosynthesis in upregulated genes. Down regulated genes in sensitive leaves on the other hand showed GO enrichment for processes like photosynthesis, ATP biosynthesis as well as ion transport (Fig. 6A). The GO names associated with these stress-induced DEGs are shown in Fig. 6B. Here, genes correlated to cellular degeneration with GO names like apoptosis, chlorophyll and PSII catabolism and proteolysis were shown to be upregulated. Moreover, ATP and chlorophyll biosynthesis were downregulated, leading to major perturbation in energy metabolism in leaves of sensitive progenies under stress. Pathway involvement of the upregulated and downregulated genes is shown in Fig. 6D. Interestingly, the OsRCI2-10 hydrophobic protein LTI6A whose gene expression was shown to be high in the roots of tolerant progenies under control treatment, was found to be upregulated only in the leaves of sensitive progenies under salt stress. A protein homologous to the OsRCI2-10 hydrophobic protein LTI6A, called OsRCI2-6 hydrophobic LTI6B, was similarly upregulated only in the sensitive phenotypes under salt stress (Supplementary Dataset 3). Both these proteins show response to multiple abiotic signals like salt, abscisic acid, cold and water deprivation. Therefore, it appears that the basal level of OsRCI2-10 hydrophobic protein LT16A involved in regulating membrane potential is high in tolerant phenotypes, even without stress.

Roots of sensitive progenies show GO enrichment only in their downregulated genes in processes related to transcription and biosynthesis (Fig. 6A). This seems to be an attempt at response to stress because GO names associated with upregulated genes include those for phosphoinositide signaling, abscisic acid biosynthesis and sulphur metabolism and transport (Fig. 6B). However, GO names for downregulated genes like signal transduction, auxin-mediated signaling, electron transport chain and DNA repair reflects that sensitive roots are unable to successfully respond to the applied stress. A greater number of glutathione-S-transferases were associated with downregulated genes in the sensitive population, again indicating lack of response to stress (Supplementary Dataset 3). Interestingly, the RAD51 DNA repair protein, found high at a basal level in sensitive roots in control treatment was downregulated under salt stress.

### Motif enrichment analyses

We did motif enrichment analyses for genes in both leaf and root tissues which responded to salinity stress. In leaf tissue, 10 motifs were enriched. These enriched motifs have transcription factor (TF) binding sites for EDT1, GT1, NTL9, CCA1, DOF.24, AHL12, ATHB12, AG, KAN1 andSPL8. EDT1 is involved in abiotic stress tolerance mechanisms, GT1 is a light response element, NTL9 is a biotic stress related motif, CCA1 is involved in regulation of the circadian rhythm, DOF.24 is a zinc finger protein associated in defense response, AHL12 regulates transcription, ATHB12 is involved in cell differentiation, AG regulates sequence specific DNA binding activity, KAN1 is involved in organ morphogenesis and SPL8 plays an essential role in the anther development process. In root tissue, 5 enriched motifs were observed in genes responding to salinity stress. These enriched motifs have TF binding sites for HAT1, SEP3, MYC3, DOF5.7 and MYB59. HAT1 is involved in chromatin silencing, SEP3 is involved in plant development, MYC3 is an activator of jasmonate regulated programs, DOF5.7 is involved in guard cell differentiation and MYB59 responds to ethylene, jasmonic acid and cadmium.

We also conducted motif enrichment analyses using DEGs from tolerant stress vs tolerant control and sensitive stress vs sensitive control categories from both tissues separated by upregulated and downregulated genes. In upregulated genes of tolerant plants, two motifs were found enriched corresponding to 4 known TF binding sites. These TF binding sites are SOC1, PI, AGL15 and MYB84. SOC1 is involved in flowering time control, PI controls flower development, AGL15 is involved in negative regulation of flowering and MYB84 is a regulator of Axillary meristem (Supplementary Info, Table 1). Moreover, the upregulated unique genes in sensitive plants showed 3 enriched motifs which corresponded to 7 different TF binding sites. These TF binding sites were SOC1, PI, SEP3, ABI4, ERF1, MYB84 and SPL14 (Supplementary Info, Table 1). SPL14 and ERF1 are involved in plant development process whereas ABI4 (abscisic acid insensitive 4 mediates abscisic acid and cytokinin inhibition of lateral root formation) acts as a transcription inhibitor for the photosynthesis related genes and SEP3 is involved in floral organogenesis (Supplementary Info, Table 1). In addition, downregulated genes in tolerant leaf tissues had no enriched motifs in their unique DE genes list but in sensitive ones, 2 motifs were over-represented. These 2 motifs were found to be similar to 5 unique TF binding sites and these were SOC1, PI, AGL15, MYB84 and SPL14 (Supplementary Info, Table 1).

Two motifs were found enriched in the tolerant upregulated root tissues which corresponded to 6 unique TF binding sites. These TF binding sites were SOC1, PI, SPL14, ABI4, ERF1 and CDC5. The CDC5 is an essential element for plant innate immunity (Supplementary Info, Table 1). On the other hand, 3 enriched motifs were found from the upregulated genes list in sensitive roots. Those 3 motifs were found common with 5 unique TF binding sites and these were SOC1, PI, AGL15, MYB84 & CDC5 (Supplementary Info, Table 1). The motif from the downregulated genes in tolerant plants corresponded with SOC1, Squamosa and ID1 TF binding sites whereas the motif from sensitive plant matched with SOC1, PI and ID1 TF binding sites.

## Discussion

Tolerant rice landraces such as Pokkali, Nona Bokra and Horkuch are essential in breeding for development of salt tolerant high yielding plants to ensure food security under changing climatic conditions. These breeding targets will likely be reached more quickly with a clear understanding of the physiological mechanisms used by these rice landraces and the underlying genes involved in conferring salt tolerance, especially, as these are likely complex traits controlled by a combination of multiple genes^23^. It is therefore important to discover the heritable combination of genes and their expression in such donors and their progenies that will allow grain setting under salt stress. The unique features of low loss in fertility and yield of Horkuch under reproductive stage stress^26^ led to the idea of developing a reciprocally crossed population with Horkuch and the high yielding but sensitive rice, IR29, in order to determine the gene expression differences leading to tolerant or sensitive phenotypes of the segregating progenies. In our studies, tolerance or sensitivity was defined by the thousand grain weight of progenies under stress.

Plant abiotic stress tolerance may be driven by different physiological responses that are partitioned across differing tissue types. For example, root tissue directly absorbs minerals and water from the soil and plays a key role in transporting resources to the leaves. In the context of salt tolerance, roots are the first site of defence by directly limiting or excluding sodium uptake. In our experiment, the functional variation in gene expression in major tissues like leaf and root were divergent and therefore may represent unique strategies related to salt tolerance or susceptibility. When progenies from reciprocal cytoplasmic parents were analysed for transcript abundance, the most notable GO categories from leaf tissue was response to stimuli in addition to cellular ketone metabolism, cellular amine metabolism, protein transport, cellular protein metabolic processes, monovalent cation transport as well as photosynthesis. Cellular ketone metabolism may be linked to leaf epidermal response to stress for cuticle formation^34^. Cellular amine metabolism, particularly under stress was reported to be associated with polyamine perturbation, which in turn may modulate homeostasis of ROS. In order to be able to produce grains under stress, leaves will need to continue photosynthesis while regulating antioxidant systems as well as suppressing ROS production. In root tissues, GO enrichments also included those for response to stimulus, defence responses, response to chemicals, programmed cell death, cellular carbohydrate metabolism, and small molecule synthesis and gene expression control. In salt enriched soils, rice roots are the first to encounter salinity stress^35^. Hence small molecule synthesis such as NO is important for inducing ABA in roots and subsequent signalling for leaf stomatal closure via increase in MAPK^36^.

The tolerant parent Horkuch and the sensitive parent IR29 have been compared in terms of genes counts with the tolerant and sensitive progenies from the reciprocally crossed populations. In both control and stressed conditions, Horkuch and the tolerant plants are highly correlated, with correlation coefficients in control and stress conditions of 0.9006 and 0.8078, respectively (Supplementary Info, Fig. 6A). Similarly, the sensitive parent IR29 and sensitive progenies were also highly correlated. In control and stressed conditions, the correlation coefficients between IR29 and sensitive progenies were 0.9195 and 0.7507, respectively (Supplementary Info, Fig. 6B). This comparative analysis thus showed that the transcript pattern under control and stress in the reciprocally crossed populations are closely associated with the tolerant and sensitive parents. Interestingly, the Horkuch♀ progenies showed greater number of both upregulated and downregulated genes in both leaf and root under stress. Annotation of the GO terms like K^+^ ion transport in Horkuch♀ leaf showed the upregulation of a number of K^+^ uptake permeases of the KUP family (Supplementary Dataset 3). K^+^ retention in leaf mesophyll mediated by KUP/HAK transporters has been shown to confer tolerance in contrasting sensitive and tolerant barley genotypes^37^. Moreover, HAK/KUP/KT have been shown to be involved in plant adaptive responses to salinity, drought and flooding^38^. On the other hand, the IR29♀ progenies exhibited upregulation of only a single voltage-gated potassium ion channel. (Specific accession number obtained from Supplementary Dataset 3 and functional annotation found at www.membrane.transport.org). Among the GO names uniquely upregulated in Horkuch♀, there were several under ‘response to stress’. GO annotation of ‘response to stress’ showed the upregulation of an ASR-like protein (Abscisic Stress-Ripening) as well as metallothionein. ASR1 proteins were first reported to be upregulated in the tolerant rice landrace, Pokkali^9^. More recently at least 5 different ASR proteins (1–5) have been cloned from tomato and ASR1 was shown to confer salt tolerance^39^. Ectopic expression of a metallothionein gene (OsMT1e-P) from the salt tolerant landrace, Pokkali, was shown to protect tobacco against salt-induced oxidative stress by scavenging ROS species^40^. A number of AN-1 like and A20/AN-1 domain-containing genes were annotated under ‘response to stress’ in Horkuch♀ progenies. An A20/AN-1 gene was also shown to confer salt tolerance to Arabidopsis^41^. Unique GO names upregulated in the IR29♀ were those of cell-adhesion, cellular cell wall re-organization, chromatin re-organization and photosynthesis.

GO processes related to ATP synthesis, such as photosynthesis and oxidative phosphorylation were downregulated in the leaf of Horkuch♀ progenies, while the IR29♀ ones showed downregulation only of the carotenoid biosynthesis process. H^+^ -ATPase activity is needed by plants to maintain a negative membrane potential in order to cope with abiotic stresses, such as salinity. However, this activity comes with an energy cost, and genotypes may need to allocate a larger ATP pool to fuel such a pump, at the expense of other metabolic processes. However this allocation will come with a yield penalty, resulting in a classical tolerance versus yield dilemma^38^. It remains to be seen, whether tolerant Horkuch♀ progenies, which are a combination of both Horkuch and IR29 nuclear genotypes, reallocate their ATP and pay a yield cost.

Upregulated genes in roots of Horkuch♀ progenies had several unique GO names, including two-component signal transduction systems (TCS or phosphorelay). Phosphorylation, which is mediated by TCS or His-to-Asp phosphorelays, is a key mechanism for stress signal transduction in cells. TCS components have been systematically identified and analyzed in Arabidopsis and rice, where there is increasing evidence that the TCS pathways are involved in response to environmental stimuli^42^. Moreover, multi-step phosphorelay two-component systems have been shown to impact tolerance against dehydration stress in wheat^43^.

The sensitive progenies respond to the stress treatment by upregulating as well as downregulating around twice the number of genes in both leaves and root compared to the tolerant ones. Thus the energy requirements for sensitive progenies are much more than in tolerant ones. Moreover, under stress, there is an extra requirement for energy to maintain metabolic homeostasis. However, sensitive plants failed to meet this requirement under stress given their downregulation of a number of major components of photosynthesis apparatus as well as that of the ATP biosynthetic process. Moreover, there were also DEGs unique to sensitive plants, such as upregulation of the chlorophyll and the PSII complex catabolic process. The GO names in sensitive progenies also included transcription factors as well as ‘response to stress’. The OsRCI2-10 Hydrophobic protein LTI6A and the OsRCI2-6 Hydrophobic protein LTI6B were upregulated under stress in leaves of the sensitive progenies whereas the former was upregulated without treatment in tolerant roots. Moreover, there was a unique upregulation of membrane potential associated genes in tolerant roots. RCI2A and RCI2B proteins are highly hydrophobic small conserved family of proteins located in the plasma membrane but whose function is not known. A possible role for AtRCI2 proteins in maintaining membrane function and/or integrity when plants have to adapt to any environmental condition that reduces water availability has been suggested^44^. Since tolerant plants express these proteins even without treatment, it might provide constitutive protection against stress such as water deprivation by preserving membrane structure. Under the GO name ‘response to stress’, sensitive plants showed upregulation of two universal stress protein domain containing proteins or USP domain proteins. Genes encoding proteins that contain the conserved 140–160 residues Universal Stress Protein (USP) domain (Pfam Accession: PF00582) are known to provide bacteria, archaea, fungi, protozoa, and plants with the ability to respond to a plethora of environmental stresses^45^. However, these have been shown to be upregulated in all genotypes of pigeon pea differing in levels of drought tolerance^46^. In this study tolerance was defined by higher grain weight under stress and it is possible that high levels of this protein may divert metabolic energy away from yields. In contrast to sensitive progenies, tolerant ones showed upregulation of an aquaporin and an AN1-like zinc finger domain containing protein. Upregulation of the aquaporin, which was found homologous to the Tonoplast Intrinsic Protein or TIP (MSU annotation 7) has been linked to cell expansion which may be very important under water deprivation conditions^47^. The upregulated AN1-like zinc finger protein in tolerant plants was observed to be homologous to multiple stress-responsive zinc-finger protein (MSU annotation 7). It has been shown that AN1-like zinc finger-containing stress associated proteins like OsSAP1/11 interact with A20 Zn-finger and receptor-like cytoplasmic kinase OsRLCK253 for conferring salt tolerance to transgenic Arabidopsis^41^. AN1/A20-like Zinc finger family proteins are evolutionarily conserved regulatory components in eukaryotic signaling circuits. In addition, the physiological functions of plant A20/AN1-ZnF family proteins have been implicated in various abiotic and biotic stress responses^48^.

Several genes under the GO-name, G-protein coupled receptor protein signaling pathway were uniquely upregulated only in roots of tolerant plants. A direct role for G-protein signaling in plant growth response to salt stress has been demonstrated in loss of function mutants of Arabidopsis. From their results, authors have proposed a model for growth promotion and attenuation of senescence by G activation during salt stress probably by relieving endoplasmic reticulum stress^49^.

## Conclusion

This study provides a resource for global analysis of gene expression patterns during an important developmental stage in salt tolerant and sensitive plants. Tolerant and sensitive F_3_ progenies were defined by yield parameters under stress at the reproductive stage and two tissue types were used for analyses, root and flag leaf. This publicly available gene atlas will facilitate the use of this data for researchers querying gene expression patterns linked to salt tolerance and various biological processes. Additionally, by comparing gene expression patterns we were able to identify differences and potentially causal genes for altered salinity stress tolerance mechanism derived from the landrace Horkuch. The expression data was validated using qPCR with two selected genes showing significant variation between sensitive and tolerant progenies under stress compared to no stress condition. In the RNAseq experiment one of the selected genes, Aquaporin (LOC_Os03g05290) showed higher upregulated expression under stress in tolerant plants compared to the sensitive progenies and the other gene OsRC12-6 (LOC_Os05g04700) showed higher upregulated expression under stress in sensitive plants compared to the tolerant ones. In the qPCR experiment the expression values showed similar result for both genes (Supplementary Info, Fig. 4A and B).

Our analysis from sensitive and tolerant progenies revealed that while enhancement in signaling is a hallmark of tolerant roots, downregulation of photosynthesis related and ATP biosynthesis genes are unique to leaves of sensitive plants. Furthermore, certain proteins were shown to be constitutively expressed in roots of tolerant plants, whose predicted function is regulation of membrane potential. This may be a crucial mechanism for protection against salt stress as well as for activation of enhanced signaling mechanisms. However, it’s a challenge separating adaptive responses from downstream consequences. In this case, QTL mapping may help address these difficulties. We also have performed QTL analysis (unpublished work) of the reciprocal population at F_2_ in an attempt to link the genetic mapping with expression responses to better understand both mechanisms and variation that could be used in breeding programs.

## Methods

### Development of reciprocal crossed populations

The inbred rice cultivars Horkuch (IRGC 31804) and IR29 (IRGC 30412) were obtained from the IRRI Gene bank. Reciprocally crossed F_1_ seeds were produced between October and November, 2011. F_1_ plants were initially confirmed by the SSR marker RM493 and F_1_s were advanced to the F_2_ generation by selfing. Over a 1000 F_2_ plants were produced in each cross-direction and a 100 from each were selected randomly and further advanced to F_3_ by single seed descent. A graphical representation detailing the development of these populations and phenotype selection is shown in Fig. 2. Here, RNAseq data of F_3_ progenies derived from Horkuch × IR29 are referred to as Horkuch♀ and those from the reciprocal cross as IR29♀.

### Categorization of sensitive and tolerant plants at reproductive stage

Reproductive stage screening under salinity stress (NaCl stress at 6 dSm^−1^) was performed for Horkuch♀ and IR29♀ F_3_ families in soil by following standard IRRI protocols^50^. The stress was applied prior to panicle initiation at 30 days after transplanting and continued until seeds were mature. F_3_ progenies from each F_2_ plant (designated as a specific F_3_ family) were considered as replicates during screening (Fig. 2). The following phenotypes were measured at maturity: spikelet fertility, panicle length, flag leaf length, 1000-grain weight (GW), total GW, grain yield, seed length and seed breadth. F_3_ families were categorized as either tolerant or sensitive based on the average 1000 GW of 5 F_3_ progenies at 6 dSm^−1^ salt stress. The 1000 GW component of crop yield indicates tolerance to stress because the plant can set grains and advance to a further generation, despite application of stress, in contrast to sensitive plants which set fewer and thinner seeds if at all. Moreover, 1000 GW has been shown to be a major determining factor for plant yield^51^. Plants were grouped as tolerant and sensitive from the two extreme tails of the distribution plot of 1000 GW (Fig. 1).

### RNA Isolation and seed weight measurement

Based on our initial categorization, we performed a second experiment focused on tolerant and susceptible families. Fifteen F_3_ experimental plants, each representing one segregating F_2_ individual were selected from each of the tolerant and sensitive categories and from each cross-direction in 2 groups, one to serve as control and the other under stress. Therefore, there were a total of 242 samples for RNAseq (2 phenotypes × 2 directions × 2 treatments (salt/control) × 15 biological replicates = 120 samples × 2 tissues = 240 + 2 parents (×2 control/stress, ×2 tissues) = 248 − 6 missing samples from control tissues = 242. Number of individuals and sequencing counts of the samples is shown in supplementary Dataset 1. For each plant, flag leaf and roots were collected separately. The plants were individually grown in Yoshida culture solution in pails covered by flat bowls with sieves though which the rice was grown^52^ in a net house at the University of Dhaka, Bangladesh. As the rice plants grew taller, the culms were supported by surrounding them with pebbles placed on top of the sieves. Bamboo columns with wide wire fencing were also placed at regular intervals among the pails to prevent plants from toppling over. Selected individuals from F_3_ families were planted randomly following incomplete block design (balanced) to avoid possible environmental and directional bias. Temperature and humidity recorded at that period averaged 27 °C at night and 34.9 °C at day. The pH of the Yoshida culture solution was adjusted daily and maintained at 5.5. The hydroponic solutions were changed at three day intervals. During the reproductive stage, when flag leaves emerged, salt stress (15 dSm^−1^ NaCl) was applied gradually in 5 dSm^−1^ increments per day. Samples (both root and flag leaf) were collected at 72 h after completion of the salt (NaCl) stress application. Samples (both root and flag leaf) from control treatment were also collected at the same time points. RNA was isolated using TRIZOL following manufacturer’s protocol (Invitrogen, USA) and quantified using Nanodrop^®^ spectrophotometer ND‐1000 (Thermo Fisher Scientific inc.). RNA samples were shipped to the University of Texas at Austin using RNAstable^®^ following the manufacturer’s protocol (Biomatrica, USA). RNAs were treated with Dnase I (Promega, USA) at a concentration of 1 unit/μg of total RNA. Total RNA purity and degradation were checked on 1% agarose gels before proceeding for RNA-seq library construction.

After tissue collection for RNAseq, plants were kept in the respective culture solution until grains were set from all panicles. Seed weights were evaluated to cross-check with the results obtained from the soil experiments described above. It has been reported that specific Na sensing takes place within seconds of its entry^53^, so that the transcript patterns observed in the current study are most likely those which were expressed after adjustment to the Na stress in both leaf and root.

### Library preparation and sequencing

To estimate genome-wide gene expression, each RNA sample was processed for short-read sequencing using an mRNA tag-seq approach developed by Meyer *et al*.^31^ and extended to the Illumina platform by Des Marais *et al*.^54^. In brief, this protocol enriches the cDNA pool for 300–500 bp fragments at the 3′ end of transcripts, and therefore, focuses sequencing effort on a smaller portion of transcripts compared to traditional whole-RNA Illumina preparations. The protocol has been used previously to evaluate tissue specific expression patterns and to measure plant responses to soil drying^30^. Validation studies have shown that read counts from this library preparation show very high correspondence with estimates of transcript abundance from q/RT-PCR. In the current study, 1 μg of total RNA for each sample were used in the mRNA-Seq library construction following the protocol described by Meyer, *et al*.^31^ at UT, Austin. According to the protocol, RNAs were first heat fragmented to remove biases resulting from variations in transcript length. Then, first strand cDNAs were synthesized using a modified oligo-dT primer aiming at 3ʹends. Prepared cDNAs were later amplified with sample-specific oligonucleotide barcodes, then quantified and pooled prior to sequencing.

Twenty-four barcodes from Illumina were used in this study for library preparation. For a single lane, 24 samples were pooled for sequencing on the Illumina Hiseq-2500 as 1 × 100 bp reads at the University of Texas at Austin’s Genome Sequencing and Analysis Facility (GSAF). Each lane (12 lanes total) of ~24 samples generated an average of ~140 million raw reads. The average sequencing depth of leaf and roots samples was ~6.6 and ~6.5 million, respectively (Supplementary Dataset 1). A small number of poor libraries (6 samples from control tissues: details mentioned above) resulted in low read counts (n = less than 500,000 raw reads) and were subsequently omitted from further studies.

### Data Processing

Quality of the raw reads were initially evaluated using FastQC^55^ and a variety of quality metrics were utilized for filtering or retaining reads. Filtering or trimming occurred when reads exhibited homopolymer stretches or polyA counts greater than 20 or 10% of the read length, respectively. Reads with average sequence quality >20, fewer than 5 unknown bases, and with a minimum of 30 bp in final length were retained. K-mer profiling with available rice transcriptomes was completed to determine the minimum cut-off length for implementation in our filter. These analyses revealed that about 90% of rice transcripts are unique at 30 bp length (Supplementary Info, Fig. 1).

Filtered libraries were mapped against the genome of Nipponbare, a cultivar of the japonica subspecies of *Oryza sativa*; using the bowtie/1.0.0 short read aligner fixing the maximum mismatches at 1 (value ranges from 1–3). We mapped 242 sequenced samples including both leaf and root tissues (Supplementary Dataset 1). The mapping efficiency varied from ~89–95% among the samples. Unmapped reads were likely poorly sequenced, containing many missing values (n’s) and unannotated gene sequences. Uniquely mapped reads were refined by samtools flag ‘bq’ at 10. We observed that some reads were aligned at a poor quality score. These were removed by using “bq” cut-off value of 10. This filtering removed another 8–9% of the aligned reads as they were not uniquely mapped to the reference. Rice annotation file (*Oryza sativa)* from Phytozome V.9^56^ was used to generate count files from the sequenced RNA data.

### Differential Gene counts and modelling in SAS JMP genomics

After generating count files from the RNAseq reads, the data were primarily filtered to remove transcripts with low counts (sum of counts less than 100 across samples). Filtered samples were KDMM normalized using JMP Genomics 7.0 (SAS, Cary NC) and further filtered by removing rows with 40% zero counts. However, KDMM normalization could not normalize the outliers present in the experiment. So, to avoid bias due to those outliers, a rarefaction curve was plotted (Supplementary Info, Fig. 2) with samples and their transcript counts. The curve showed that samples with a minimum of 100 k filtered reads captured ~70% of the genes occurring in the transcript pool. Therefore, samples with fewer than 100 k read counts were removed and not used for the differential gene count analysis. Despite this filtering, the remaining samples provide a good representation of replicates from each category of the experimental design (Supplementary Dataset 1). This filtering resulted in ~13,500 transcripts for leaf samples and ~14,900 transcripts for root ones. A simple generalized linear mixed model was fitted for normalized count data for each transcript using a negative binomial distribution and a log link function. The model included factors for cytoplasm (IR29♀ or Horkuch♀), treatment (salt stress or control), and phenotype (tolerant or sensitive). Pairwise interactions included treatment × cross direction and treatment × phenotype, along with a random effect for sequencing lane. Given their complexity, higher-order interactions were not considered but rather pooled in the residual of the model. The false-discovery rate (FDR) was set as 0.05 in the study using the Benjamin-Hochberg method. The expression data was validated using qPCR with two selected genes showing significant variation between sensitive and tolerant progenies under stress compared to no stress condition. For the qPCR experiment and analysis, Biorad CFX96^TM^ realtime thermocycler and CFX96 manager software were utilized using LightCycler^®^480 SYBR green master mix. Two technical replicates and four biological replicates were used for the Tolerant and Sensitive category across control and stressed samples (Supplementary Info, Fig. 4A and B). A putative proteasome subunit gene (LOC_Os03g63430) in rice was used as internal control.

### GO annotation

Differentially expressed genes from all fixed and contrast models were evaluated for gene-set enrichments by AgriGO^57^ using a hypergeometric test after Hochberg FDR correction with a significance level of p < 0.05. Later, GO enrichment sets were further summarized using ReviGO by removing redundant GO names. For ReviGO analysis, the *Oryza sativa* database was selected for the GO names, where SimRel was used as a standard for semantic similarity measurement. We also used the Rice Oligonucleotide Array Database for further GO association studies^58^. A comparative analysis was made between the GO names of different experimental categories. For example, tolerant upregulated leaf DEGs were compared with sensitive upregulated leaf and so on. We focused on DEGs on the basis of their association with salt stress by mining literature. Specific details are provided in the relevant supplementary excel files, where the first sheet provides the detailed workflow, including the title of the contents of each sheet (Supplementary Datasets 2 and 3).

### Motif identification and enrichment analysis

We retrieved 1000 bp sequences upstream of coding regions of selected gene lists using Biomart V0.7 from the tools window of Phytozome 10.2 Genomes database^56^. Motif identification and enrichment analysis was performed using MEME-ChIP prediction tool^59^. In this analysis, JASPAR CORE Plantae was the database used for motif search. Tomtom^60^ was used to compare the motif-motif similarity between our datasets and those of JASPAR CORE Plantae.

## Additional Information

**Accession codes:** RNA-seq data analyzed here has been deposited in NCBI database under BioProject ID: PRJNA317262. SRA submission id: SRP090879 and can be accessed at https://trace.ncbi.nlm.nih.gov/Traces/study/?acc=SRP090879.

**How to cite this article**: Razzaque, S. *et al*. Reproductive stage physiological and transcriptional responses to salinity stress in reciprocal populations derived from tolerant (Horkuch) and susceptible (IR29) rice. *Sci. Rep.* **7**, 46138; doi: 10.1038/srep46138 (2017).

**Publisher's note:** Springer Nature remains neutral with regard to jurisdictional claims in published maps and institutional affiliations.

## Supplementary Material

Supplementary Information

Supplementary Dataset 1

Supplementary Dataset 2

Supplementary Dataset 3

## Figures and Tables

**Table 1:** Number of differential Genes from reproductive leaf and root tissues in different models and interactions.

Comparison	Fixed models/interactions	DEGS (n) in Leaf Samples (down/up regulated)	DEGs (n) in Root samples (down/up regulated)
Cross direction	IR29♀ vs Horkuch♀	↓664 ↑231	↓158 ↑131
Treatment	Stress vs Control	↓802 ↑1399	↓665 ↑816
Phenotype	Tolerant vs Sensitive	↓21 ↑9	↓9 ↑11
Cross direction × Treatment	Horkuch♀ Stress vs Horkuch♀ Control	↓733 ↑997	↓435 ↑600
	IR29♀ Stress vs IR29♀ Control	↓271 ↑644	↓267 ↑259
Phenotype × Treatment	Sensitive Stress vs Sensitive Control	↓228 ↑428	↓185 ↑278
	Tolerant Stress vs Tolerant Control	↓139 ↑272	↓173 ↑193

## References

[b1] Gupta B & Huang B. Mechanism of salinity tolerance in plants: physiological, biochemical, and molecular characterization. Intl. J. Genomics 2014, Article ID 701596, 18 pages, doi: 10.1155/2014/701596 (2014). PMC3996477 24804192

[b2] Haque M., Jahiruddin M., Hoque M., Rahman M. & Clarke D. Temporal variability of soil and water salinity and its effect on crop at Kalapara upazila. J. Environ. Sci. Nat. Res. 7, 111–114 (2015).

[b3] Lisa L. A. . Genetic variation in microsatellite DNA, physiology and morphology of coastal saline rice (*Oryza sativa* L.) landraces of Bangladesh. Plant and Soil 263, 213–228 (2004).

[b4] Walia H. . Comparative transcriptional profiling of two contrasting rice genotypes under salinity stress during the vegetative growth stage. Plant Physiol. 139, 822–835 (2005). 16183841 10.1104/pp.105.065961 PMC1255998

[b5] Khan M., Hamid A. & Karim M. Effect of sodium chloride on germination and seedling characters of different types of rice (*Oryza sativa* L.). J. Agro. Crop Sci. 179, 163–169 (1997).

[b6] Moradi F., Ismail A. M., Egdane J. & Gregorio G. B. & Salinity tolerance of rice during reproductive development and association with tolerance at the seedling stage. Ind. J. Plant Physiol. 8, 105–116 (2003).

[b7] Moradi F. & Ismail A. M. Responses of photosynthesis, chlorophyll fluorescence and ROS-scavenging systems to salt stress during seedling and reproductive stages in rice. Ann. Bot. 99, 1161–1173 (2007). 17428832 10.1093/aob/mcm052 PMC3243573

[b8] Walia H. . Genome-wide transcriptional analysis of salinity stressed japonica and indica rice genotypes during panicle initiation stage. Plant Mol. Biol. 63, 609–623 (2007). 17160619 10.1007/s11103-006-9112-0 PMC1805040

[b9] Kawasaki S. . Gene expression profiles during the initial phase of salt stress in rice. The Plant Cell 13, 889–905 (2001). 11283343 10.1105/tpc.13.4.889 PMC135538

[b10] Fukuda A. . Function, intracellular localization and the importance in salt tolerance of a vacuolar Na(+)/H(+) antiporter from rice. Plant Cell Physiol. 45, 146–159 (2004). 14988485 10.1093/pcp/pch014

[b11] Wu S., Ding L. & Zhu J. SOS1, a genetic locus essential for salt tolerance and potassium acquisition. The Plant Cell 8, 617–627 (2003). 10.1105/tpc.8.4.617 PMC161124 12239394

[b12] Ren Z.-H. . A rice quantitative trait locus for salt tolerance encodes a sodium transporter. Nature Genet. 37, 1141–1146 (2005). 16155566 10.1038/ng1643

[b13] Parvin S. . Salinity and drought tolerance conferred by in planta transformation of SNAC1 transcription factor into a high-yielding rice variety of Bangladesh. Acta Physiol. Plant. 37, 1–12 (2015).

[b14] Ying S. . Cloning and characterization of a maize bZIP transcription factor, ZmbZIP72, confers drought and salt tolerance in transgenic Arabidopsis. Planta 235, 253–266 (2012). 21866346 10.1007/s00425-011-1496-7

[b15] Karaba A. . Improvement of water use efficiency in rice by expression of HARDY, an Arabidopsis drought and salt tolerance gene. Proc. Natl. Acad. Sci. 104, 15270–15275 (2007). 17881564 10.1073/pnas.0707294104 PMC1986572

[b16] Amin M. . Over-expression of a DEAD-box helicase, PDH45, confers both seedling and reproductive stage salinity tolerance to rice (*Oryza sativa* L.). Mol. Breeding 30, 345–354 (2012).

[b17] Singla-Pareek S. L., Reddy M. K. & Sopory S. K. Genetic engineering of the glyoxalase pathway in tobacco leads to enhanced salinity tolerance. Proc. Nat. Acad. Sci. 100, 14672–14677 (2003). 14638937 10.1073/pnas.2034667100 PMC299757

[b18] Xu D. . Expression of a Late Embryogenesis Abundant Protein Gene, HVA1, from Barley Confers Tolerance to Water Deficit and Salt Stress in Transgenic Rice. Plant Physiol. 110, 249–257 (1996). 12226181 10.1104/pp.110.1.249 PMC157716

[b19] Nakashima K. & Yamaguchi‐Shinozaki K. Regulons involved in osmotic stress‐responsive and cold stress‐responsive gene expression in plants. Physiol. Plantarum 126, 62–71 (2006).

[b20] Blumwald E., Aharon G. S. & Apse M. P. Sodium transport in plant cells. BBA-Biomembranes 1465, 140–151 (2000). 10748251 10.1016/s0005-2736(00)00135-8

[b21] Roy S. J., Negrao S. & Tester M. Salt resistant crop plants. Curr. Opin. Biotechnol. 26, 115–124 (2014). 24679267 10.1016/j.copbio.2013.12.004

[b22] Pires I. S., Negrao S., Oliveira M. M. & Purugganan M. D. Comprehensive phenotypic analysis of rice (*Oryza sativa*) response to salinity stress. Physiol. Plant. 155, 43–54 (2015). 26082319 10.1111/ppl.12356

[b23] Thomson M. J. . Characterizing the Saltol quantitative trait locus for salinity tolerance in rice. Rice 3, 148–160 (2010).

[b24] Garcia A., Senadhira D., Flowers T. J. & Yeo A. R. The effects of selection for sodium transport and of selection for agronomic characteristics upon salt resistance in rice (*Oryza sativa* L.). Theor. Appl. Genet. 90, 1106–1111 (1995). 24173070 10.1007/BF00222929

[b25] Anil V. . Regulation of the uptake and distribution of Na^+^ in shoots of rice (*Oryza sativa*) variety Pokkali: role of Ca^2+^ in salt tolerance response. Physiol. Plantarum 124, 451–464 (2005).

[b26] Lisa L. . Physiology and gene expression of the rice landrace Horkuch under salt stress. Funct. Plant Biol. 38, 282–292 (2011). 10.1071/FP10198 32480884

[b27] Dionisio-Sese M. L. & Tobita S. Effects of salinity on sodium content and photosynthetic responses of rice seedlings differing in salt tolerance. J. Plant Physiol. 157, 54–58 (2000).

[b28] Ismail A. M., Heuer S., Thomson M. J. & Wissuwa M. Genetic and genomic approaches to develop rice germplasm for problem soils. Plant Mol. Biol. 65, 547–570 (2007). 17703278 10.1007/s11103-007-9215-2

[b29] He G. . Global epigenetic and transcriptional trends among two rice subspecies and their reciprocal hybrids. The Plant Cell 22, 17–33 (2010). 20086188 10.1105/tpc.109.072041 PMC2828707

[b30] Lovell J. T. . Promises and challenges of eco-physiological genomics in the field: tests of drought responses in switchgrass. Plant physiol., pp. 00545.02016 (2016). 10.1104/pp.16.00545 PMC5047078 27246097

[b31] Meyer E., Aglyamova G. & Matz M. Profiling gene expression responses of coral larvae (*Acropora millepora*) to elevated temperature and settlement inducers using a novel RNA-Seq procedure. Mol. Ecol. 20, 3599–3616 (2011). 21801258 10.1111/j.1365-294X.2011.05205.x

[b32] Shankar R., Bhattacharjee A. & Jain M. Transcriptome analysis in different rice cultivars provides novel insights into desiccation and salinity stress responses. Sci. Rep. 6 (2016). 10.1038/srep23719 PMC4814823 27029818

[b33] Moons A. . An abscisic-acid- and salt-stress-responsive rice cDNA from a novel plant gene family. Planta 202, 443–454 (1997). 9265787 10.1007/s004250050148

[b34] Samuels L., Kunst L. & Jetter R. Sealing plant surfaces: cuticular wax formation by epidermal cells. Ann. Rev. Plant Biol. 59, 683–707 (2008). 18251711 10.1146/annurev.arplant.59.103006.093219

[b35] Julkowska M. M. . Capturing Arabidopsis root architecture dynamics with ROOT-FIT reveals diversity in responses to salinity. Plant Physiol. 166, 1387–1402 (2014). 25271266 10.1104/pp.114.248963 PMC4226346

[b36] Yu M., Lamattina L., Spoel S. H. & Loake G. J. Nitric oxide function in plant biology: a redox cue in deconvolution. The New Phytologist 202, 1142–1156 (2014). 24611485 10.1111/nph.12739

[b37] Wu H., Zhu M., Shabala L., Zhou M. & Shabala S. K+ retention in leaf mesophyll, an overlooked component of salinity tolerance mechanism: a case study for barley. J. Integr. Plant Biol. 57, 171–185 (2015). 25040138 10.1111/jipb.12238

[b38] Shabala S., Bose J., Fuglsang A. T. & Pottosin I. On a quest for stress tolerance genes: membrane transporters in sensing and adapting to hostile soils. J. Exp. Bot. 67, 1015–1031 (2016). 26507891 10.1093/jxb/erv465

[b39] Golan I. . Tomato Abscisic Acid Stress Ripening (ASR) gene family revisited. PLoS One 9, e107117 (2014). 25310287 10.1371/journal.pone.0107117 PMC4195575

[b40] Kumar G. . Clustered metallothionein genes are co-regulated in rice and ectopic expression of OsMT1e-P confers multiple abiotic stress tolerance in tobacco via ROS scavenging. BMC Plant Biol. 12, 107 (2012). 22780875 10.1186/1471-2229-12-107 PMC3491035

[b41] Giri J., Vij S., Dansana P. K. & Tyagi A. K. Rice A20/AN1 zinc-finger containing stress-associated proteins (SAP1/11) and a receptor-like cytoplasmic kinase (OsRLCK253) interact via A20 zinc-finger and confer abiotic stress tolerance in transgenic Arabidopsis plants. The New phytologist 191, 721–732 (2011). 21534973 10.1111/j.1469-8137.2011.03740.x

[b42] Mochida K. . Genome-wide analysis of two-component systems and prediction of stress-responsive two-component system members in soybean. DNA research: Intl. J. Rapid Pub. Rep. Genes Genomes 17, 303–324 (2010). 10.1093/dnares/dsq021 PMC2955714 20817745

[b43] Gahlaut V. . A multi-step phosphorelay two-component system impacts on tolerance against dehydration stress in common wheat. Funct. Integr. Genomics 14, 707–716 (2014). 25228409 10.1007/s10142-014-0398-8

[b44] Medina J., Ballesteros M. L. & Salinas J. Phylogenetic and functional analysis of Arabidopsis RCI2 genes. J. Exp. Bot. 58, 4333–4346 (2007). 18182435 10.1093/jxb/erm285

[b45] Isokpehi R. D. . Identification of drought-responsive universal stress proteins in viridiplantae. *Bioinformatics Biol*. Insights 5, 41–58 (2011). 10.4137/BBI.S6061 PMC3045048 21423406

[b46] Sinha P. . Identification and Validation of Selected Universal Stress Protein Domain Containing Drought-Responsive Genes in Pigeonpea (*Cajanus cajan* L.). Front. Plant Sci. 6, 1065 (2015). 26779199 10.3389/fpls.2015.01065 PMC4701917

[b47] Maurel C., Verdoucq L., Luu D. T. & Santoni V. Plant aquaporins: membrane channels with multiple integrated functions. Annu. Rev. Plant Biol. 59, 595–624 (2008). 18444909 10.1146/annurev.arplant.59.032607.092734

[b48] Zhang Y. . An A20/AN1-type zinc finger protein modulates gibberellins and abscisic acid contents and increases sensitivity to abiotic stress in rice (*Oryza sativa*). J. Exp. Bot. 67, 315–326 (2016). 26512055 10.1093/jxb/erv464

[b49] Colaneri A. C., Tunc-Ozdemir M., Huang J. P. & Jones A. M. Growth attenuation under saline stress is mediated by the heterotrimeric G protein complex. BMC Plant Biol. 14, 129 (2014). 24884438 10.1186/1471-2229-14-129 PMC4061919

[b50] Gregorio G. B., Senadhira D. & Mendoza R. D. Screening rice for salinity tolerance. *IRRI Disc*. Paper Series No. 22 (1997).

[b51] Ishimaru K. . Loss of function of the IAA-glucose hydrolase gene TGW6 enhances rice grain weight and increases yield. Nature Genet. 45, 707–711 (2013). 23583977 10.1038/ng.2612

[b52] Yoshida S., Forno D., Cock J. & Gomez K. Routine procedure for growing rice plants in culture solution. Lab. Manual Physiol. Studies Rice, 61–66 (1976).

[b53] Ismail A., Takeda S & Nick P. Life and death under salt stress: same players, different timing? J. Exp. Bot. eru159 (2014). 10.1093/jxb/eru159 24755280

[b54] Des Marais D., Skillern W. & Juenger T. Deeply Diverged Alleles in the Arabidopsis AREB1 Transcription Factor Drive Genome-Wide Differences in Transcriptional Response to the Environment. Mol. Biol. Evol. 32 956–969 (2015). 25540452 10.1093/molbev/msu401

[b55] Andrews S. FastQC: A quality control tool for high throughput sequence data. *Reference Source* http://www.bioinformatics.babraham.ac.uk/projects/fastqc/ (15 October 2015, date last accessed) (2010).

[b56] Goodstein D. M. . Phytozome: a comparative platform for green plant genomics. Nucleic Acids Res. 40, D1178–D1186 (2012). 22110026 10.1093/nar/gkr944 PMC3245001

[b57] Du Z., Zhou X., Ling Y., Zhang Z. & Su Z. agriGO: a GO analysis toolkit for the agricultural community. Nucleic Acids Res. gkq310 (2010). 10.1093/nar/gkq310 PMC2896167 20435677

[b58] Cao P. . The Rice Oligonucleotide Array Database: an atlas of rice gene expression. Rice (N Y) 5, 17 (2012). 24279809 10.1186/1939-8433-5-17 PMC4883718

[b59] Machanick P. & Bailey T. L. MEME-ChIP: motif analysis of large DNA datasets. Bioinformatics 27, 1696–1697 (2011). 21486936 10.1093/bioinformatics/btr189 PMC3106185

[b60] Bailey T. L., Johnson J., Grant C. E. & Noble W. S. The MEME Suite. Nucleic Acids Res. gkv416 (2015). 10.1093/nar/gkv416 PMC4489269 25953851

