# Supplementary material for: Reproductive stage physiological and transcriptional responses to salinity stress in reciprocal populations derived from tolerant (Horkuch) and susceptible (IR29) rice
Source: Sci Rep. 2017 Apr 11;7:46138. doi: 10.1038/srep46138 (PMC5387399; doi:10.1038/srep46138)
Supplement: Supplementary Information [file srep46138-s1.doc]

**Title:** Reproductive stage physiological and transcriptional responses to salinity stress in reciprocal populations derived from tolerant (Horkuch) and susceptible (IR29) rice

**Authors:** Samsad Razzaque^1,2†^, Taslima Haque^1,2†^, Sabrina M Elias^1,5†^, Md. Sazzadur Rahman^3^, Sudip Biswas^1^, Scott Schwartz^2^, Abdelbagi M Ismail^4^, Harkamal Walia^5^, Thomas E Juenger^2^ and Zeba I Seraj^1*^

**Supplementary Figures**


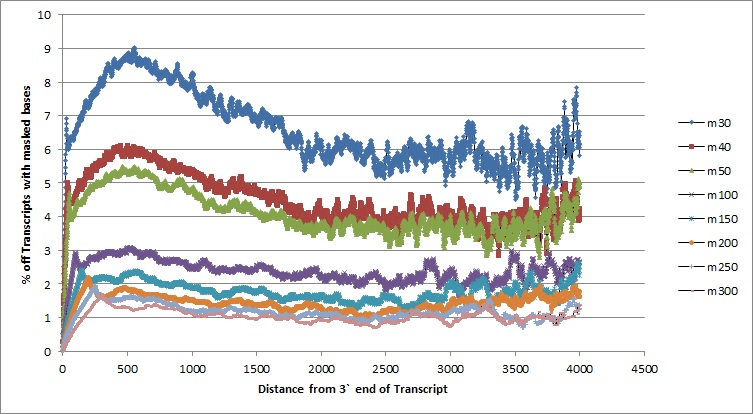


**Supplementary Fig. 1:** k-mer profiling with available rice transcriptome data. It shows that 90% of rice transcripts can be uniquely captured using 30bp transcript sequence

**
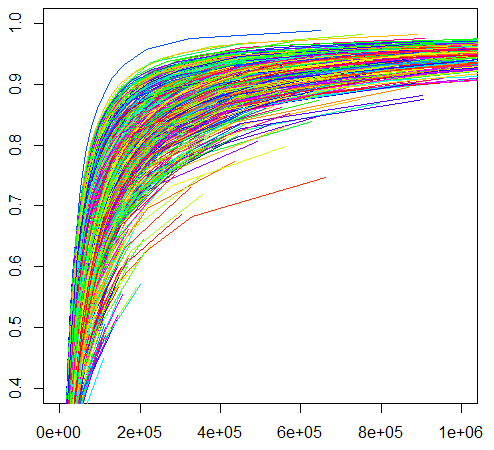
**

**Supplementary Fig. 2:** A rarefaction curve shows that samples with a minimum of 100k filtered reads captured ~70% of the transcript counts.


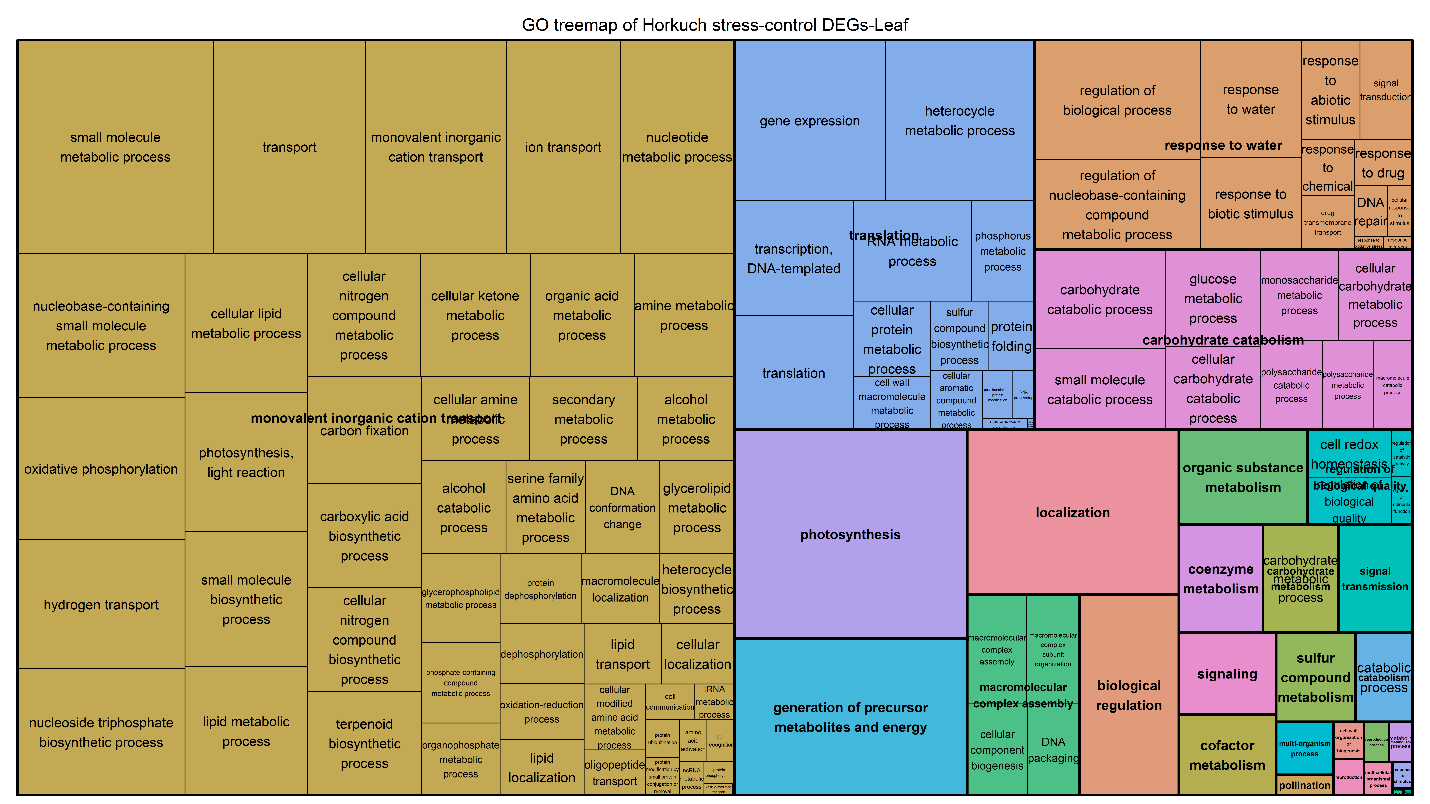


**Supplementary Figure 3A**

**
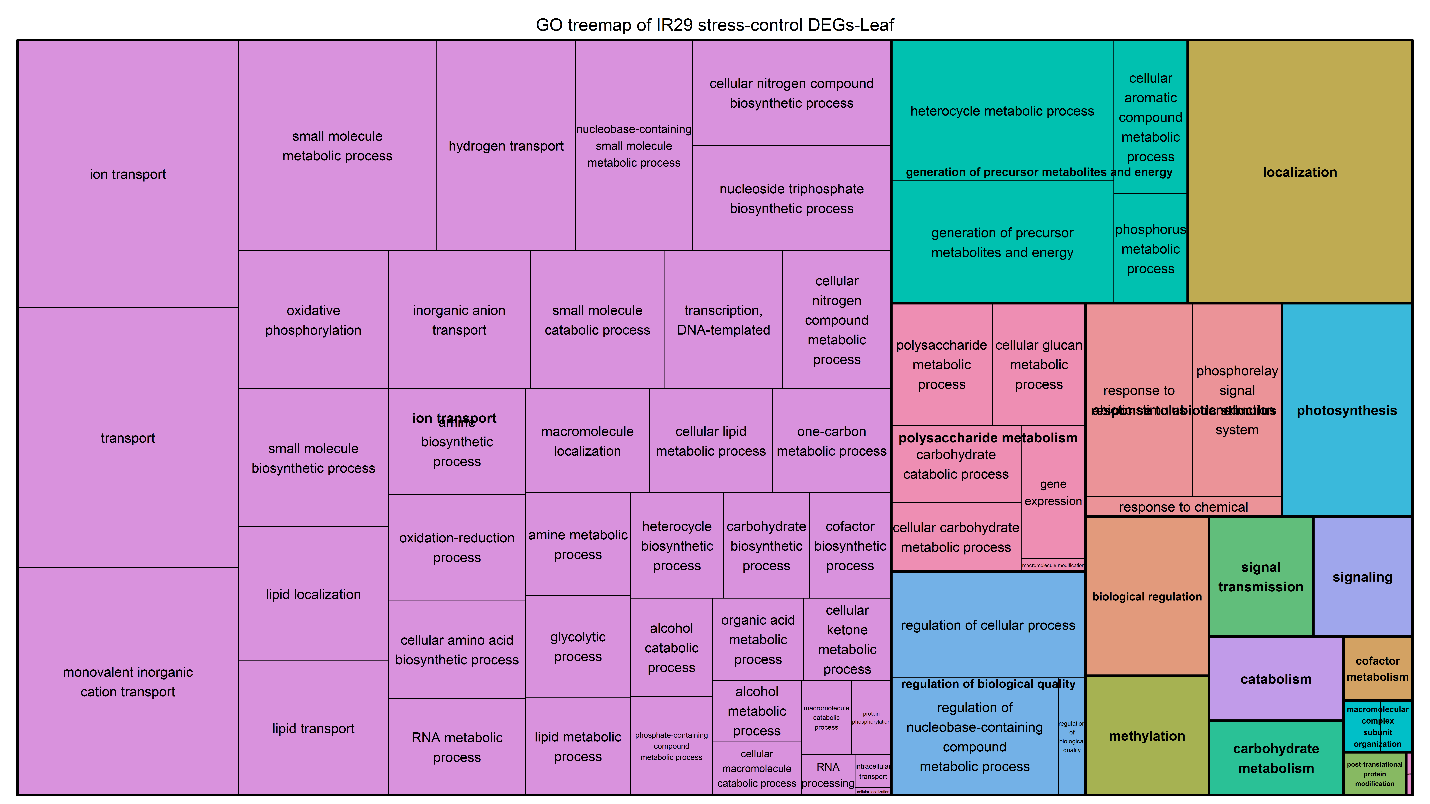
**

**Supplementary Figure 3B**


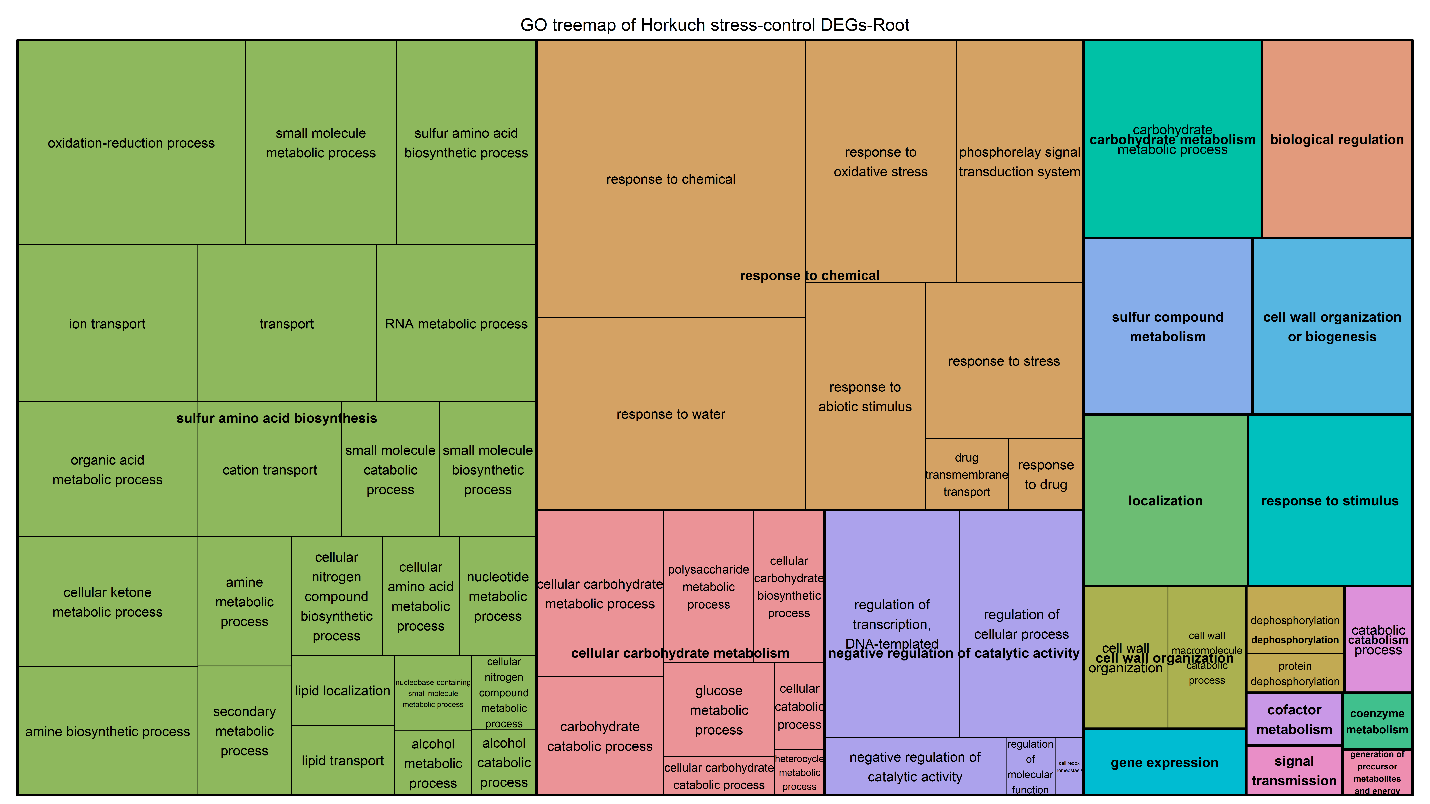


**Supplementary Figure 3C**


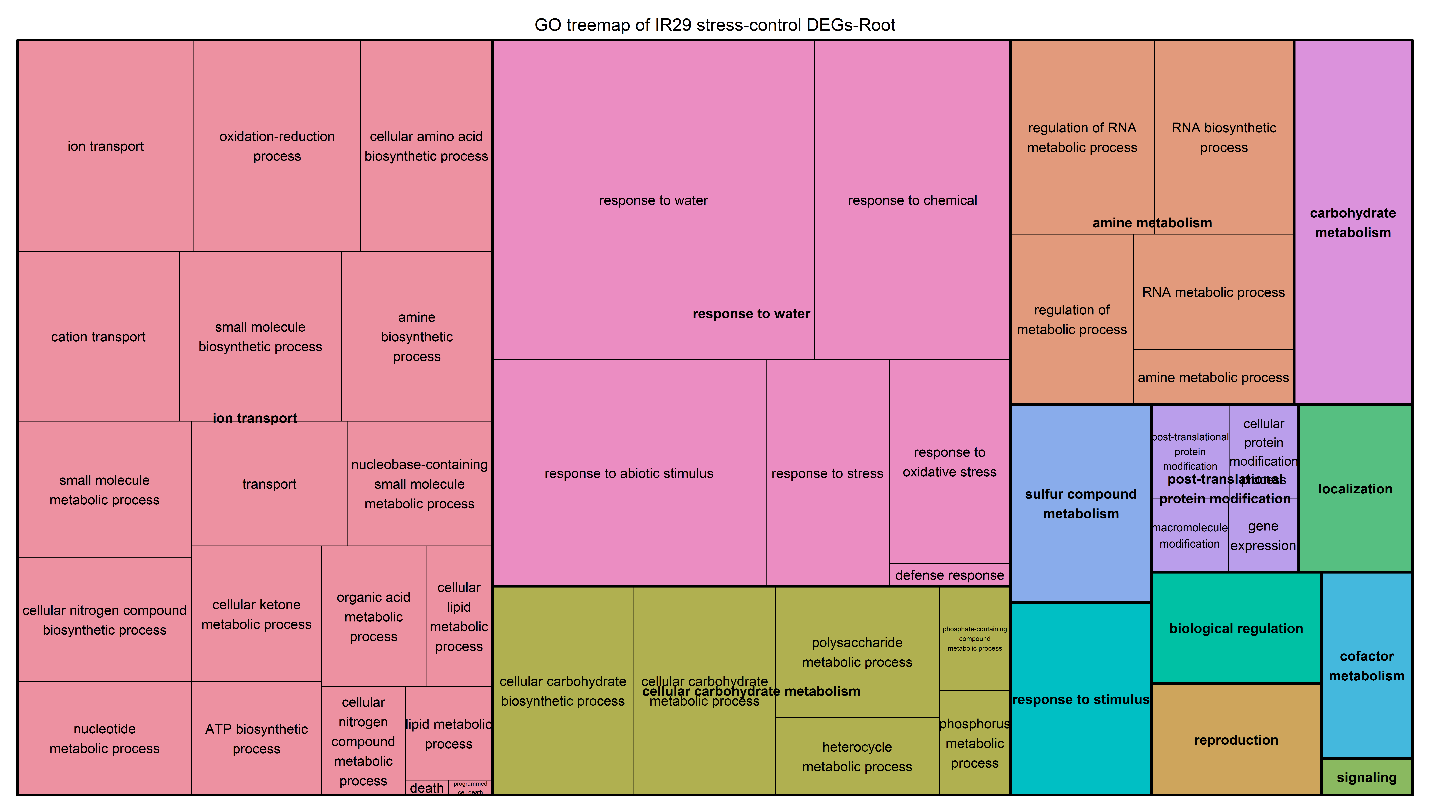


**Supplementary Figure 3D**

**Supplementary Fig. 3:** Gene Ontology treemap for the 1% highly expressed genes. The box size correlates to the –log10 p-value of the GO-term enrichment. Boxes with the same color can be grouped together and correspond to the same upper-hierarchy GO-term. **A and B:** denotes the treemap generated from the leaf tissues’ DEGs under cytoplasm*treatment interaction effects. The treemap shows the GO enrichment for the DE genes from Horkuch stress vs Horkuch Control and IR29 stress vs IR29 control in leaf tissues. **C and D:** shows the enriched GO names generated from root tissues’ DEGs under cytoplasm*treatment interaction effects. The treemap shows the GO enrichment for the DE genes from Horkuch stress vs Horkuch Control and IR29 stress vs IR29 control in root tissues.


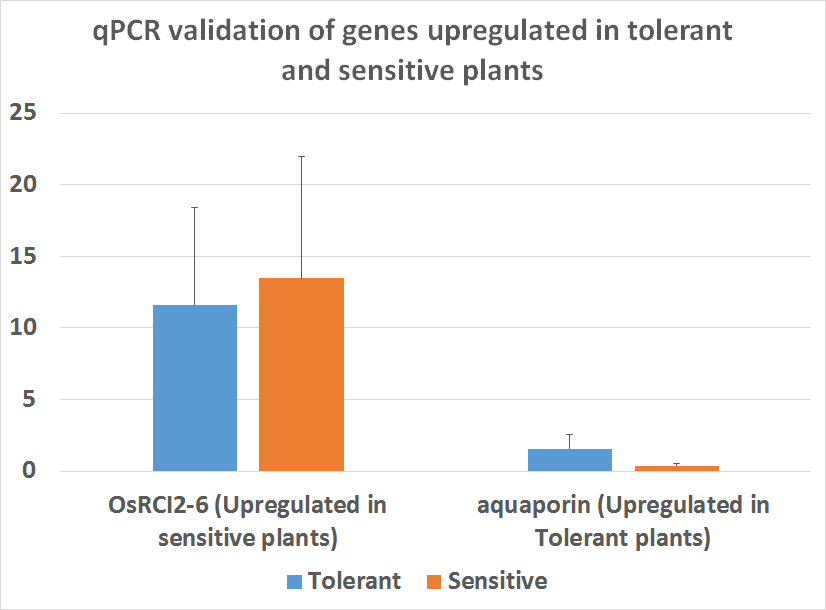


**Supplementary Fig. 4A**


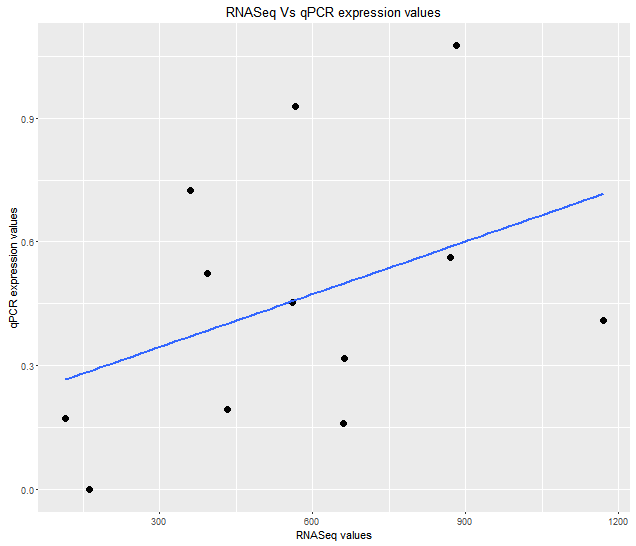


**Supplementary Fig. 4B**

**Supplementary Fig 4A:** Genes reported with upregulated expression in sensitive and tolerant progenies under stress in the RNAseq analysis also showed similar pattern of expression in the qPCR experiment. **Fig. 4B**: Overall expression trend of RNAseq and qPCR experiment expression values from both were plotted for OsRCl12-6 gene and regression line was fitted, with r^2^ = 0.16. The correlation value for the expression values from both platforms was 0.4

**
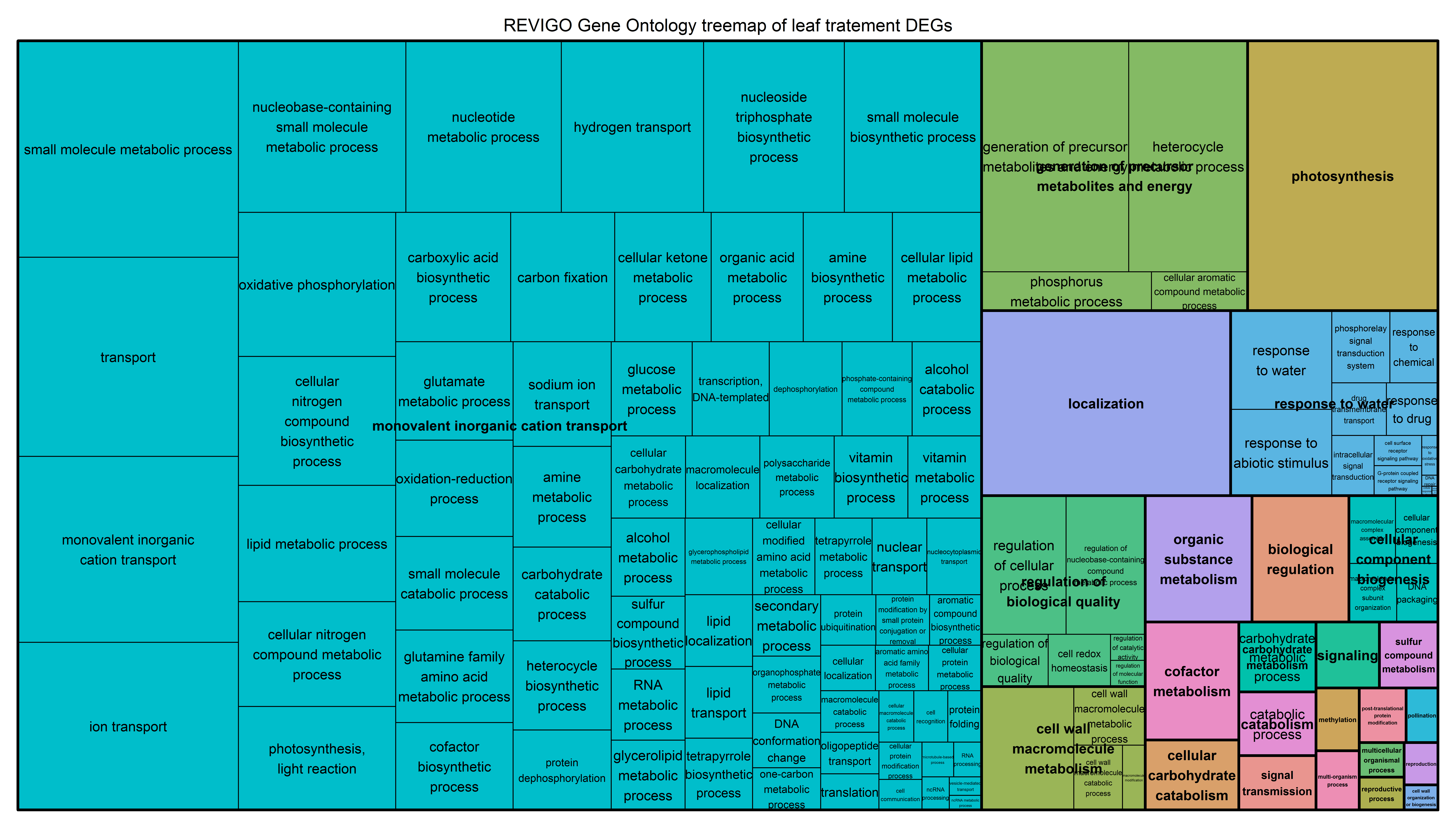
**

**Supplementary Figure 5A**

**
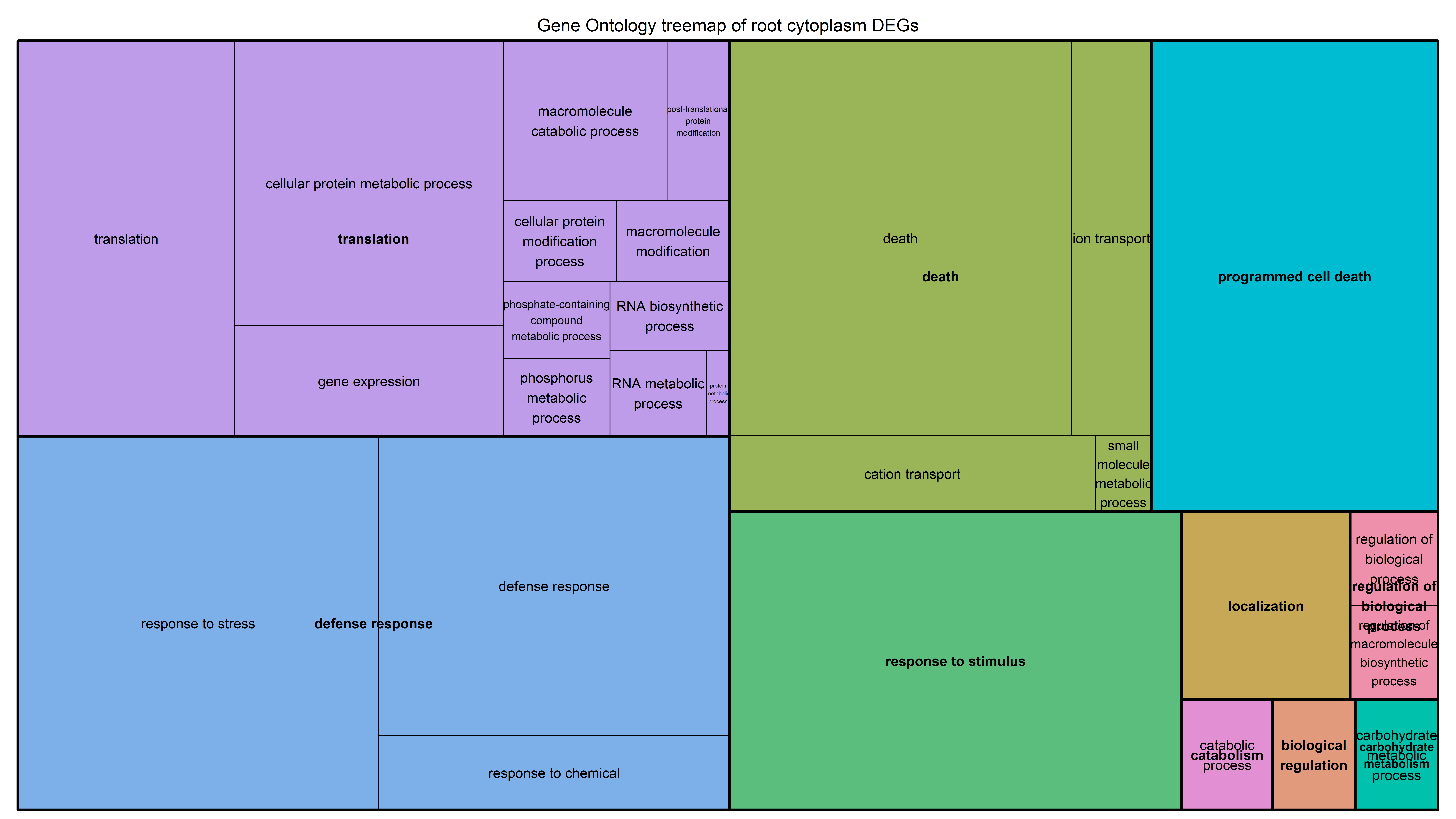
**

**Supplementary Figure 5B**

**
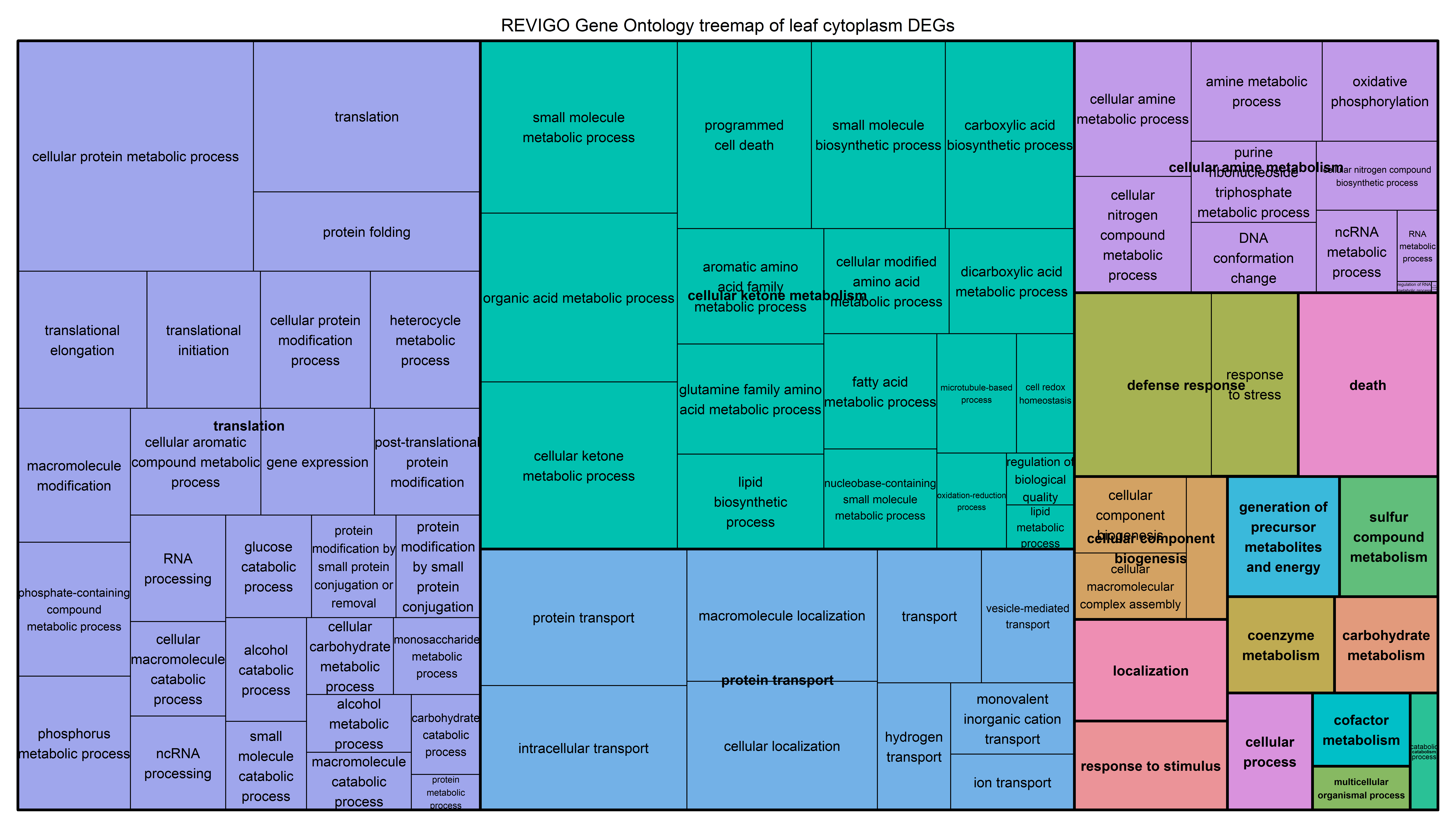
**

**Supplementary Figure 5C**

**
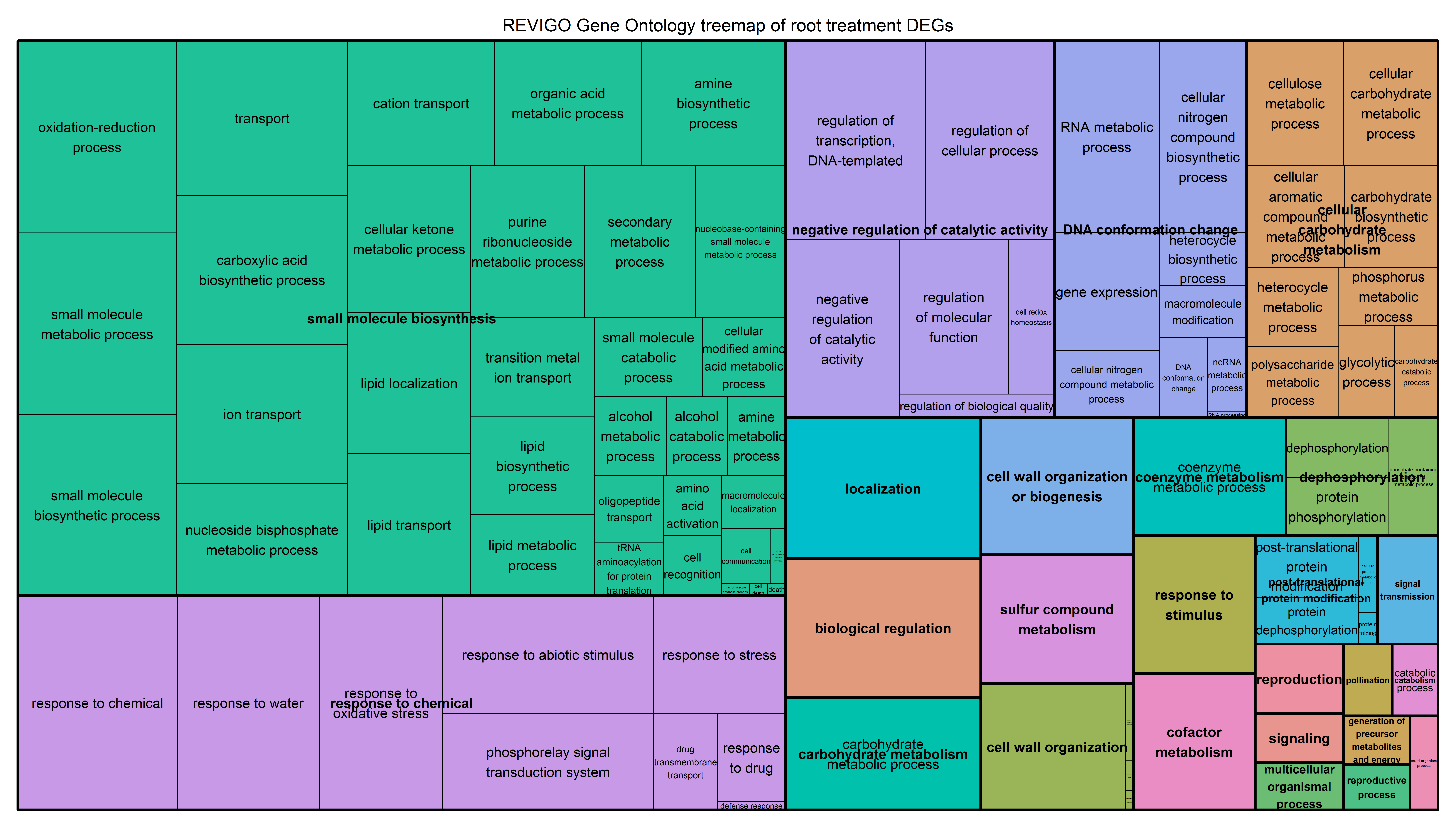
**

**Supplementary Figure 5D**

**Supplementary Fig. 5:** Gene Ontology treemap for the 1% highly expressed genes. The box size correlates to the –log10 p-value of the GO-term enrichment. Boxes with the same color can be grouped together and correspond to the same upper-hierarchy GO-term. **A and C:** denotes the treemap generated from the leaf tissues’ DEGs under cytoplasm and treatment effects. These GO enrichments are from the fixed model in leaf tissues without interactions with other experimental factors. **B and D:** shows the enriched GO names generated from root tissues’ DEGs under cytoplasm and treatment effects. These GO enrichments are from the fixed model in root tissues without interactions with other experimental factors. In these two effects cytoplasm shows the GO enrichments of the DEgenes detected from Horkuch and IR29 cross directions and the treatment shows the GO enrichments from the genes detected from control and salinity condition.

**Supplementary Figure 6A**

**Supplementary Figure 6B**

**Supplementary Fig. 6:** The Tolerant parent Horkuch and the sensitive parent IR29 have been compared with the tolerant and sensitive progenies from the reciprocally crossed populations. Fig. 6A shows the correlations between the parent and the tolerant progenies. In control and stress condition, Horkuch and the tolerant plants are highly correlated, correlation coefficients in control and stress conditions are 0.9006 and 0.8078, respectively. Fig. 6B shows the correlation between sensitive IR29 with the sensitive progenies. Like the tolerant plants, they also show high correlations. In control and stress condition, the correlation coefficients between IR29 and sensitive progenies are 0.9195 and 0.7507, respectively.

**Supplemental Materials:**

**Supplementary Table 1:** Over-represented motifs in the promoters of rice (*Oryza sativa*) genes that are upregulated or downregulated in sensitive and tolerant plants in response to salinity stress as identified using the programs MEME-ChIP and Tomtom

| DE gene list | Tissue | Regulation | Sequence | E-value | Matched to known motif |
| --- | --- | --- | --- | --- | --- |
| Sensitive stress versus sensitive control | Leaf | Down | AA[TC]A[G]AAATAA[C]AA | 4.4e-018 | SOC1, PI, AGL15 |
|  |  |  | C[T]CT[C]CCTCC[T]G[A]TCC | 1.7e-003 | MYB84,SPL14 |
| Sensitive stress versus sensitive control | Leaf | Up | AAA[G]AA[G]A[T]AAA[TT]A[GT]T[G]A[T] | 1.5e-054 | SOC1, PI, SEP3 |
|  |  |  | GG[CA]C[GA]GG[CT]CGG[A]CG[C]GC | 3.4e-011 | ABI4, ERF1 |
|  |  |  | CCC[T]CC[TA]CC[T]T[CG]CT[C]C[T]C | 9.4e-004 | MYB84, SPL14 |
| Tolerant Stress versus tolerant control | Leaf | Down | No enrichment observed |  |  |
| Tolerant Stress versus tolerant control | Leaf | Up | A[T]AA[C]AAA[G]AA[CT]T[A]AA[G] | 3.6e-040 | SOC1, PI, AGL15 |
|  |  |  | T[G]C[TG]CT[A]C[TA]CC[TA]C[TG]T[C]CC[T] | 3.5e-002 | MYB84 |
| Sensitive stress versus sensitive control | Root | Down | A[G]TT[CA]T[GA]T[CA]T[C]TTTT[CA]T[G]A[TC] | 1.3e-013 | SOC1, Squamosa, id1 |
| Sensitive stress versus sensitive control | Root | Up | A[TG]A[G]A[C]AA[G]AAAA[TG]A[T]A[C] | 1.4e-015 | SOC1, PI, AGL15 |
|  |  |  | CC[A]C[AG]CC[G]CC[G]C[T]G[A]C | 6.7e-005 | MYB84, CDC5 |
|  |  |  | AT[CG]G[A]GAG[T]G[A]GAG[C]T[A]A | 2.4e-002 | SOC1, PI, AGL15 |
| Tolerant stress versus tolerant control | Root | Down | A[G]AA[CG]A[T]AG[A]AA[G]AA[G]A[G] | 1.0e-003 | SOC1, PI, id1 |
|  |  | Up | A[G]AAA[TG]AA[TC]AAA[C]A[T]T[GA] | 1.5e-009 | SOC1, PI, Squamosa |
|  |  | Up | GC[G]C[G]G[A]C[T]C[G]G[C]C[G]C[A]C[T]GC | 7.8e-004 | ERF1, cdc5, abi4 |

**Supplementary File Legends**

**Supplementary File 1:** Experimental samples described by number and identity of samples, sequencing counts, cross direction, treatment and phenotype (**Data file uploaded as the Dataset 1**).

**Supplementary File 2:** GO mapping of unique DEGs in sensitive and tolerant population.  GO mapping (Biological Process) of DEGs in leaf and root are shown in two separate worksheets of the supplementary excel file. Uniquely up and down-regulated genes and associated GOs are separately listed (**Data file uploaded as the Dataset 2**).

**Supplementary File 3:** Comparison of GO names and metabolic pathways associated with unique up- and down-regulated DEGs in leaf and root with respect to cross-direction and phenotype. Sheet 1 shows the workflow as well as the location in sheet number of the different DEGs contrasting genotypes in subsequent sheets (**Data file uploaded as the Dataset 3**).
